# Strengthening implementation science research to improve adolescent and young adult HIV-prevention and care in Sub-Saharan Africa: PATC^3^H-IN

**DOI:** 10.1186/s12889-025-24995-0

**Published:** 2025-11-26

**Authors:** Geri R. Donenberg, Ucheoma Nwaozuru, Sylvia Adebajo, Joseph D. Tucker, Juliet Iwelunmor, Lisa M. Kuhns, Brenda Gati Mirembe, Lisa Hightow-Weidman, Brian Zanoni, Tapiwa A. Tembo, Maria Felix Lupogo, Franklin Yates

**Affiliations:** 1 Center for Dissemination and Implementation Science, Department of Medicine, University of Illinois Chicago, Chicago, IL USA; 2 Department of Implementation Science, Wake Forest University School of Medicine, Winston-Salem, NC USA; 3 Institute of Human Virology, University of Maryland, Baltimore, USA; 4 Institute of Human Virology, Abuja, Nigeria; 5 Clinical Research Department, Faculty of Infectious and Tropical Diseases, London School of Hygiene and Tropical Medicine, LSHTM, London, England; 6 Institute for Global Health and Infectious Diseases, University of North Carolina at Chapel Hill, Chapel Hill, NC USA; 7 Division of Infectious Diseases, Washington University School of Medicine in St. Louis, Saint Louis, MO USA; 8 Division of Adolescent Medicine, Ann & Robert H. Lurie Children’s Hospital, Chicago, IL USA; 9 Department of Pediatrics, Feinberg School of Medicine, Northwestern University, Chicago, IL USA; 10 MU-JHU Research Collaboration, Kampala, Uganda; 11 College of Nursing, Florida State University, Florida, USA; 12 Department of Pediatrics, Emory University School of Medicine, Atlanta, GA USA; 13 Baylor College of Medicine Children’s Foundation Malawi, Lilongwe, Malawi; 14 Health for a Prosperous Nation, Dar es Salaam, Tanzania; 15 Eunice Kennedy Shriver National Institute of Child Health and Human Development, National Institutes of Health, DC, USA

**Keywords:** Adolescents and young adults, Implementation science, HIV prevention and care, Evidence-based interventions, Africa

## Abstract

**Background:**

Despite significant progress in HIV prevention and treatment, uptake of evidence-based interventions among adolescents and young adults (AYA), particularly in low- and middle-income countries (LMICs), remains low. Implementation research can optimize strategies to enhance reach, uptake, and equitable access to these innovations. The Prevention and Treatment through a Comprehensive Care Continuum for HIV-affected Adolescents in Resource-Constrained Settings Implementation Science Network (PATC^3^H-IN) leverages implementation science to strengthen the delivery and sustainability of evidence-based HIV prevention and care for AYA across six countries in sub-Saharan Africa. This paper outlines PATC^3^H-IN’s goals, summarizes the implementation science (IS) data that will be collected, and highlights the advantages of research networks in advancing science.

**Methods:**

The PATC^3^H-IN builds on the existing PATC^3^H consortium to advance IS research targeting AYA in LMICs. The PATC^3^H-IN comprises eight Clinical Research Centers (CRCs) located in Nigeria, Uganda, Malawi, South Africa, Zambia, and Tanzania. Representatives from the CRCs were asked to provide information on the IS components of their proposed studies, including details on study populations, IS frameworks, outcomes, and strategies, mechanisms of change, effectiveness outcomes, and documentation of intervention adaptations. The reports from the CRCs were compared to identify opportunities for advancing science across study sites.

**Results:**

The PATC^3^H-IN studies will enroll AYA aged 12–24 years, with some emphasizing key subpopulations, namely AYA living with HIV, sexual and gender minorities, and adolescent girls and young women. All PATC^3^H-IN studies will be guided by one or more implementation science frameworks and theories, with the Reach, Effectiveness, Adoption, Implementation, and Maintenance (RE-AIM) framework most frequently cited (*n* = 4/8). Across the CRCs, 54 unique implementation strategies will be used, with community engagement being the most common. Several studies will document intervention adaptations, and all studies will collect a set of common data elements to facilitate secondary data analyses across projects.

**Conclusions:**

The PATC^3^H-IN represents a significant contribution to advancing HIV prevention and care research for AYA in resource-constrained settings. Findings from PATC^3^H-IN will extend our understanding of IS in sub-Saharan Africa, a region particularly burdened by HIV, and for AYA who are traditionally under-represented in IS research.

**Clinical trial number:**

Not Applicable.

**Supplementary Information:**

The online version contains supplementary material available at 10.1186/s12889-025-24995-0.

## Background

Tremendous achievements have been made since the first AIDS diagnosis in 1981, transforming HIV from an untreatable disease to a fully preventable chronic condition [1]. Scientific advancements have led to effective biomedical approaches that could end the epidemic, including formulations that minimize side effects, reduce patient burden, and remain effective for longer periods of time [2]. Similarly, new behavioral tools have strengthened prevention [2, 3] and improved HIV testing uptake, linkage and retention in care, and adherence to antiretroviral therapy (ART) [4, 5]. Yet, many of the world’s most vulnerable populations have not benefitted equally from these innovations, in large part a consequence of minimal attention to contextual factors, structural determinants, and system-level barriers at each stage of the HIV-prevention and care continuum, particularly in resource-constrained settings [6]. 

Implementation science (IS) holds great promise in improving the uptake and adoption of life-saving interventions, but only when evidence-based interventions are tailored for the specific culture, context, and population, engage key partners and communities, employ strategies that address population-specific barriers, and target change mechanisms [7]. Unfortunately, efforts to transfer knowledge across situations and settings have been largely disappointing, particularly from high-income to low-to-middle-income environs, between LMICs, and across regions, countries, and implementing partners within sub-Saharan Africa, where the largest number of people have HIV and where adolescents and young adults (AYA) ages 15–24 represent the group at greatest risk of new infections. Despite the high burden of HIV among AYA in this region, HIV testing rates, levels of ART initiation, viral suppression, retention in care, and ART adherence are lower among AYA compared to adults. According to the United Nations, sub-Saharan Africa has the youngest population in the world, with 70% of the population under 30 years old, raising alarms about the future if current HIV incidence rates persist [8]. The reasons for the low uptake of evidence-based HIV interventions in lower-income settings are complex, but frequently implicated is the absence of attention to unique contextual barriers, facilitators, and constraints [9–11]. Improving uptake and adoption of evidence-based interventions in sub-Saharan Africa is, therefore, among the most important public health challenges if we hope to end HIV as a public health threat.

In direct response to this shifting landscape, *The Eunice Kennedy Shriver* National Institute of Child Health and Human Development (NICHD) launched the first *Prevention And Treatment through a Comprehensive Care Continuum for HIV-affected Adolescents in Resource Constrained Settings* (PATC^3^H) consortium comprised of eight research projects implemented across six countries in sub-Saharan Africa and Brazil (see Tucker, Iwelunmor, Abrams, et al., 2021 [12], for a full description). Briefly, the original PATC^3^H network focused on implementing and evaluating interventions to achieve long-term viral suppression among AYA with HIV or to prevent HIV acquisition among AYA who engage in behaviors that place them at risk for HIV [12]. The studies utilized innovative combination multilevel interventions to improve AYA health outcomes and to explore implementation-related factors that constrained and/or facilitated evidence-based intervention delivery [13]. In 2023, NICHD built on the progress and momentum of the PATC^3^H network. The new PATC^3^H-Implementation Science Network (PATC^3^H-IN) expanded the focus on implementation science and HIV-related research to prevent, diagnose, link to care, and achieve viral suppression among AYA living in sub-Saharan Africa. The PATC^3^H-IN [14] emphasizes rigorous implementation science approaches to strengthen the reach, access, adoption, affordability, scale-up, and sustainability of evidence-based interventions by localizing and capacitating research in eight Clinical Research Centers (CRC) across six low- to middle-income countries in sub-Saharan Africa. This paper describes the goals of PATC^3^H-IN identified by the eight study teams, and the cross-study implementation science characteristics, to underscore opportunities for cooperation and mutual learning best achieved through a network of projects.

Several advantages of research conducted as a network of projects go beyond the benefits of a single study. Research networks can advance public health when multiple projects test a similar scientific question simultaneously, and investigators can discuss and learn from one another about potential barriers and constraints. Research networks facilitate cross-study pollination of ideas and methodologies, allowing for innovations in real time. Additionally, discoveries can be implemented and expanded more quickly by other projects. Research networks may guide practice decision-making with greater confidence when findings generalize across populations and settings. Finally, research networks can adopt common data elements and harmonize data collection tools, thereby informing novel measurement approaches, driving the science of instrumentation forward, and contributing new findings about the specific topic being measured.

## Methods

### PATC^3^H-IN

PATC^³^H-IN comprises eight CRCs located in high HIV incidence communities across sub-Saharan Africa. The studies supported by each CRC represent a collaboration among investigators and implementers at each stage of the research process, including understanding, guiding, and evaluating the effectiveness and implementation of evidence-based strategies to prevent new HIV infections among AYA, and/or identify, link, retain, and achieve long-term viral suppression among AYA with HIV. The studies will evaluate diverse and innovative interventions (see Table 1).

**Table 1:** PATC^3^H-IN CRCs (2024–2029)

Project Name and Corresponding Acronyms	Implementing Location	Project Description
Evaluation of Long-Acting Injectable and Teen Clubs in Adolescents in South Africa (ATTUNE)	South Africa	This study aims to improve HIV care outcomes among adolescents living with HIV in South Africa by evaluating the effectiveness of peer navigation and long-acting injectable antiretroviral therapy (LA-ART). This will include the use of adolescent-friendly services and peer navigation to optimize behavioral interventions before investigating long-acting injectable antiretroviral therapy for adolescents living with HIV in sub-Saharan Africa.
Intensive Combination Approach to Rollback the HIV Epidemic in Nigerian Youth Plus Effectiveness/Implementation Hybrid Study (iCARE Plus)	Nigeria	This study aims to improve HIV prevention and treatment outcomes among Nigerian youth aged 15–24, particularly young men who have sex with men (YMSM) and young transgender women (YTW). The project will scale out and test effectiveness and implementation outcomes on enhanced versions of the iCARE interventions, which include peer navigation and mHealth strategies to increase: (a) HIV case-finding, linkage-to-care, and PrEP uptake among YMSM and YTW, and (b) viral load suppression in previously unstudied YLH newly-diagnosed and initiating ART.
Implementation Science to Understand and Design Stakeholder-Informed Innovative Interventions to Improve Adolescent and Youth HIV Prevention and Care Continuums in Rural and Urban Uganda (MUJHU)	Uganda	This study aims to evaluate evidence-null improve implementation outcomes among high-risk AYA (15–24 years) in Uganda. The study will implement context-specific community differentiated service delivery models for Cabotegravir, long-acting antiretroviral (CAB-LA) and evaluate a multi-component intervention comprising of life-stage based assessment and support to increase viral suppression in high-risk AYA with HIV.
Making Women’s Options for HIV Prevention in Tanzania Accessible and Joining Implementation Science Capacity Building (MWOTAJI)	Tanzania	This project aims to evaluate the Malkia Klabu (“Queen Club”) implementation strategy in Tanzania, a loyalty program designed for and by AGYW that creates youth-friendly pharmacies where AGYW can access HIV prevention and sexual and reproductive health (SRH) services, with strong linkages to facility-based care.
Resilient HIV Implementation Science with Sexual and Gender Minority Youths using Evidence Clinical Research Center (RISE)	Nigeria, Kenya, Malawi, Zambia	This project aims to adapt, refine, and implement a digital health platform to support HIV prevention and care continuum among sexual and gender minority youth in Nigeria, Kenya, Malawi, and Zambia.
Sustaining Innovative Tools to Expand Youth-Friendly HIV Self-Testing (S-ITEST)	Nigeria	This project aims to use participatory approaches (i.e., crowdsourcing designathons and participatory learning communities) to co-create sustainment strategies for the 4Youth By Youth (4YBY) program. The 4YBY program aims to promote the uptake of HIV self-testing and other preventive services among AYA, 14–24 years.
VITAL (Video-Intervention to Inspire Treatment Adherence for Life) Start for Adolescents (VS4A)	Malawi	This project aims to adapt, assess, and support the scale-up of a tailored, video-based counseling package and intervention to improve retention and adherence to ART among adolescents living with HIV.
Zambian Informed, Motivated Aware and Responsible Adolescents and Adults (ZAIMARA)	Zambia	This project will evaluate the effectiveness and implementation of a CDC “best-evidence” intervention, Informed Motivated Aware and Responsible Adolescents and Adults (IMARA), recently adapted for South Africa (IMARA-SA) on Zambian AGYW HIV testing uptake, STI/HIV incidence, and PrEP uptake.

### PATC^3^H-IN network goals

All PATC^3^H-IN projects were awarded for five years, beginning in October 2023. At the PATC^3^H-IN kick-off meeting in January 2024, each of the eight study teams from Nigeria (S-ITEST, iCARE Plus), Uganda (HIP-CY), Malawi (VS4A), South Africa (ATTUNE), Zambia (ZAIMARA), Tanzania (MWOTAJI), and one team representing Nigeria, Kenya, Malawi and Zambia (RISE) shared their goals for the network. Attendees discussed synergies before launching the studies. The discussions confirmed the value of implementation science (IS was built into the call for proposals, so all staff saw the value of it), building collaborations across the network (e.g., cross-training opportunities), and the development and adoption of common data elements in recognition of the importance of comparing findings across projects. Teams were represented at the meeting by principal investigators, partners, health ministers, AYA representatives, NICHD and Fogarty program staff, Westat (the NIH-contracted company to assist with logistics), and other research project members. The teams reached consensus on five main goals for the network: (1) Develop collaborations across CRCs; (2) build capacity within CRCs to cultivate the next generation of implementation science scholars; (3) strengthen the *science* of implementation science; (4) amplify the scientific impact of the PATC^3^H-IN network; and (5) establish PATC^3^H-IN as a leader in AYA HIV-prevention and care research. The CRCs also agreed on the important role of youth engagement in their projects. Table 2 describes the network’s planned activities to achieve these goals.

**Table 2:** Goals for the PATC^3^H-IN network

Goals	Activities
1) Develop collaborations across CRCs	Create complementary research initiatives Develop new networks Define ideas for joint manuscripts Establish learning collaboratives Build local, regional, and cross-country alliances Facilitate South-to-South learning
2) Build capacity within CRCs and train the next generation of implementation science scholars	Train local providers in implementation science Develop cutting-edge resources Deliver implementation science curricula across CRCs Train to use mHealth strategies Diversify the science pipeline Provide mentorship for junior scholars Elevate youth voices from development to dissemination Create a standing youth advisory board to direct network studies Provide pilot funding for sub-studies
3) Strengthen the *science* of implementation science	Link implementation strategies to health/implementation outcomes Collect implementation science measures across studies Create guidelines for co-design workshops Grow the literature on effective implementation strategies
4) Amplify PATC^3^H-IN’s scientific impact	Learn to write policy briefs Translate research findings to inform national and global guidelines Measure success and impact across the consortium Align study findings with PEPFAR initiatives and goals Leverage South-to-South alliances for larger impacts Adapt studies to different regions Strengthen strategies for scale-up
5) Be a leader in adolescent HIV prevention and care research	Minimize duplication of studies Create a data harmonization platform Pool data for subsequent analyses Increase scientific efficiency Share best practices in service delivery Expose investigators and partners to new ideas Identify new formats and technologies to strengthen health outcomes Nimbly address emerging research concerns in adolescent HIV Define how network projects address adolescent-identified concerns

*Notes: CRCs *=Clinical Research Centers; *PATC*^*3*^*H-IN=*Prevention and Treatment through a Comprehensive Care Continuum for HIV-affected Adolescents in Resource-Constrained Settings Implementation Science Network

Further, each team reported on the primary features of their research study, namely the target population, implementation theory or framework(s) guiding their project, implementation outcomes of interest, hypothesized mechanisms of change, planned implementation strategies, and any approach to documenting adaptations of interventions and/or implementation strategies. The remainder of this paper describes the implementation characteristics across the studies, concluding with a discussion about the value of a network in advancing science beyond any single individual project.

### Ethics approval

This study did not involve humans or human data. Instead, we summarized the implementation data proposed to be collected within the PATC^3^H-IN network studies. The data were obtained from responses from the PATC^3^H-IN network investigators or NIH reporter. Therefore, informed consent was not required for this study and IRB approval was not needed. However, every project presented in the paper has been approved by its local ethics group.

## Results

### Study populations

The PATC^3^H-IN network is focused on diverse populations of AYA from six countries in East, West, and Southern Africa (see Table 3). Study populations span the developmental age range from 12 to 24-years-old and represent key populations most vulnerable to HIV transmission and poor health outcomes associated with HIV. All eight studies will enroll adolescents (10–17-year-olds) and young adults (18–24 years-old). Two studies will focus exclusively on primary HIV prevention, and three studies will target youth with HIV. Three studies will include youth with and without HIV. Four studies will intentionally recruit sexual and gender minorities, and two studies will focus exclusively on adolescent girls and young women (AGYW). All studies will emphasize individual-level behavior change, but three studies will also engage the broader context to strengthen AYA outcomes. Specifically, two will engage primary caregivers (VS4A, ZAIMARA), and two will focus on healthcare facility workers and pharmacists (HIP-CY, MWOTAJI). In total, PATC^3^H-IN will enroll 15,386 adolescents and young adults, constituting one of the largest research samples of young people affected by HIV in sub-Saharan Africa.

**Table 3:** Study population characteristics

Study Name	HIP-CY	i-CARE Plus	RISE	SI-TEST	ATTUNE	ZAIMARA	VS4A	MWOTAJI
Country	Uganda	Nigeria	Nigeria Kenya Malawi Zambia	Nigeria	South Africa	Zambia	Malawi	Tanzania
Sample size	600	6600	1500	1,216	720	600 dyads (1200 ppts)	1800	1750
10–17 years-old (HIV+)	Yes	Yes	Yes	Yes	Yes	No	Yes	No
10–17 years-old (HIV-)	Yes	Yes	Yes	Yes	No	Yes	No	Yes
18–24 years- old (HIV+)	Yes	Yes	Yes	Yes	Yes	No	Yes	No
18–24 years- old (HIV-)	Yes	Yes	Yes	Yes	No	Yes	No	Yes
Key population	Yes	YMSM YWH	SGM	Not targeted; Not excluded	Not targeted; Not excluded	AGYW	Not targeted; Not excluded	AGYW
Other populations	Health care providers; Pharmacists	--	--	--	--	Mother figures enrolled with AGYW	Parents/ guardians; Health care workers	Pharmacy staff; Health facility staff; government stakeholders

*Notes: YMSM*=Young men who have sex with men; *SGM *=Sexual and gender minorities; *AGYW *=Adolescent girls and young women; *YWH *=Youth with HIV

### Implementation science theories and frameworks

All PATC^3^H-IN studies will be guided by one or more of the seven established implementation science frameworks and theories described by Nilsen’s (2015) [15] taxonomy (i.e., evaluation, determinants, and process). The most common framework that will be used is the Reach, Effectiveness, Adoption, Implementation, Maintenance (RE-AIM) evaluation framework, employed by four of the projects [16]. RE-AIM refers to four aspects of intervention implementation within health settings, namely whether it is: “reaching” the target population, “effective” at improving the clinical outcome, “implemented” with fidelity, and “maintained” in the health care system [17]. The second most common framework, the Consolidated Framework for Implementation Research (CFIR), will be employed by three studies and describes the determinants of implementation [18]. These studies will elucidate the barriers, facilitators, and constraints affecting implementation across the five CFIR domains (intervention characteristics, individual characteristics, outer setting, inner setting, and implementation processes) [19]. The CFIR will also inform study assessments of implementation outcomes (see below).

Two additional frameworks will guide network projects: the *E*xploration, *P*reparation, *I*mplementation, *S*ustainment (EPIS) framework and the Implementation Research Logic Model (IRLM). EPIS focuses on the process of implementation, describing four stages of implementation and the outer and inner context factors [20] that inform the implementation process. Research suggests that the time spent in specific EPIS phases predicts successful implementation of evidence-based interventions [21]. The IRLM was developed to strengthen the scientific specification, rigor, reproducibility, and transparency [22] of implementation science studies by specifying relationships across implementation determinants, mechanisms of change, implementation strategies, and outcomes [7]. The IRLM also helps identify the most relevant implementation strategies to employ based on certain determinants and mechanisms.

### Implementation science outcomes

All PATC^3^H-IN studies will evaluate one or more implementation outcomes based on the Proctor et al., (2011) Implementation Outcomes Framework [23]. The absence of validated tools to measure implementation outcomes in LMICs, however, has posed a challenge to the network. It is unclear if measures used in high-income countries can be applied in resource-constrained settings, or if they are missing important outcomes unique to these contexts. Building on the original PATC^3^H consortium’s data harmonization process and common data elements [13], PATC^3^H-IN investigators agreed on an expanded set of implementation outcomes and corresponding measurement approaches to use across the network. Both quantitative and qualitative measures will be used across the PATC^3^H-IN studies. Notably, all eight projects will examine *Effectiveness*, *Implementation*,* Sustainability*/*Maintenance* and *Adoption*, seven projects will assess *Reach*, six will evaluate *Feasibility*, *Acceptability*, and *Costing/cost effectiveness.* Additionally, three will measure *Appropriateness* and *Fidelity* and one will employ time and motion studies (see Table 4)for the implementation outcomes measured across the CRCs).)

**Table 4:** Implementation outcomes

Study Name	HIP-CY	i-CARE Plus	RISE	SI-TEST	ATTUNE	ZAIMARA	VS4A	MWOTAJI
Acceptability	-	Yes	Yes	Yes	Yes	Yes	Yes	-
Adoption	Yes	Yes	Yes	Yes	Yes	Yes	Yes	Yes
Appropriateness	-	Yes	-	-	-	Yes	-	Yes
Costing/Cost effectiveness	Yes	-	Yes	Yes	Yes	Yes	Yes	-
Effectiveness	Yes	Yes	Yes	Yes	Yes	Yes	Yes	Yes
Feasibility	Yes	Yes	-	Yes	Yes	Yes	Yes	-
Fidelity	Yes	-	-	-	-	Yes	Yes	-
Implementation	Yes	Yes	Yes	Yes	Yes	Yes	Yes	Yes
Reach	-	Yes	Yes	Yes	Yes	Yes	Yes	Yes
Sustainability/Maintenance	Yes	Yes	Yes	Yes	Yes	Yes	Yes	Yes

### Mechanisms of change

As the field of implementation science continues to gain traction, there is a growing emphasis on identifying the mechanisms of change [24, 25]. Identifying change mechanisms can guide the selection of implementation strategies that address the barriers, facilitators and constraints to achieve change. PATC^3^H-IN studies will evaluate multi-level mechanisms from the individual to the health system. Five studies will focus on individual-level mechanisms, such as enhancing participant motivation, increasing treatment self-efficacy, addressing mental health symptoms, and improving HIV knowledge. Four studies will address interpersonal-level mechanisms by enhancing effective communication with peers, partners, and parents/guardians, increasing healthy sexual relationships, and strengthening peer support for prevention and care. Two studies will enhance participatory and human-centered design mechanisms. Finally, three projects will emphasize health-system-level mechanisms, namely strengthening connections between clinical staff and youth and engaging pharmacy owners and staff.

### Implementation strategies

Study teams listed their planned implementation strategies, and these were categorized according to Powell’s (2015) Expert Recommendations for Implementing Change (ERIC) taxonomy [26]. The PATC^3^H-IN studies will employ a total of 54 unique implementation strategies, with study teams proposing a range of 8–37 each. Broadly, the implementation strategies are focused on three areas: community engagement, capacity support, and learning to optimize implementation. The most common implementation strategies planned by the seven study teams are community engagement and capacity support for implementation success. They will engage advisory boards and workgroups, develop educational materials, and intervene with patients/consumers to enhance uptake and adherence. The second most common implementation strategies also emphasize community collaborations, capacity building, and optimizing implementation success by attending closely to cultural context, for example, assessing for readiness, identifying barriers and facilitators, building a coalition, conducting educational meetings and local consensus discussions, and involving patients/consumers and family members. Finally, several ERIC strategies were adopted by just one project, such as centralizing technical assistance, conducting cyclical small tests of change, developing a formal implementation blueprint, promoting network weaving, shadowing other experts, and visiting other sites. Details on the planned implementation strategies are provided in Supplementary File 1.

### Effectiveness and other outcomes

All eight studies will evaluate the intervention’s effectiveness on participant outcomes (see Table 5). For studies of youth with HIV, five will assess the impact on viral suppression, three will examine retention in HIV care, and one will test linkage to care, ART initiation, and ART adherence using a combination of biological specimens and health records. Five projects of youth without HIV will evaluate PrEP uptake, and four will assess PrEP persistence. One will examine linkage to PrEP care, and another will test PrEP re-start for those who stop and begin again. Three of these projects will calculate HIV testing uptake and HIV incidence. Three studies will examine sexual behaviors that contribute to the spread of HIV, including self-reported condomless sex, multiple partners, sex with high-risk partners, and positive tests of sexually transmitted infections. All of these are indicators of risk for HIV acquisition. Importantly, all primary prevention studies will assess attitudes and/or perceptions of oral and injectable PrEP to inform future studies of uptake and adoption of long-acting regimens, including both Cabotegravir [27] and Lenacapavir [28].

**Table 5:** Effectiveness and other outcomes

HIV-Continuum	Effectiveness and Other Outcomes	Studies
HIV-care and treatment continuum	Viral suppression	ATTUNE, iCARE Plus, HIP-CY, RISE, VS4A
	Retention in care	ATTUNE, RISE, VS4A
	Linkage to care	iCARE Plus
	ART adherence	iCARE Plus, HIP-CY
	ART initiation	RISE
HIV-prevention continuum	HIV-risk behavior	ATTUNE, iCARE Plus, S-ITEST, ZAIMARA, HIP-CY
	HIV testing uptake	iCARE Plus, S-ITEST, RISE, ZAIMARA
	Linkage to PrEP	iCARE Plus, S-ITEST
	PrEP uptake	iCARE Plus, HIP-CY, RISE, SI-TEST, ZAIMARA, MWOTAJI
	PrEP persistence	HIP-CY, MWOTAJI, RISE, ZAIMARA
	PrEP restart	RISE, MWOTAJI
	HIV incidence	iCARE Plus, RISE, ZAIMARA, HIP-CY
	STI incidence	ZAIMARA

### Adaptation of evidence-based interventions and strategies

A key component of implementation science emphasizes ensuring fit between an intervention and the target population, setting, and context [29]. Accordingly, six of eight PATC^3^H-IN projects will systematically adapt (either pre- or post-launch) an evidence-based intervention to the local contexts and populations where they will be delivered (see Table 6). Most projects will undergo pre-intervention adaptations using participatory and/or user-centered approaches that include local partners (e.g., youth, adults, and community advisory boards). The most common pre-intervention adaptation framework used in three studies is the ADAPT-ITT model, a pragmatic approach to adapting interventions developed in the United States to international settings [30–34]. The eight-step framework promotes a combination of formative research to determine local capacity and available resources, plus real-time input from the target population (e.g., theater testing), partners and experts, and pilot testing to create the final intervention [35]. Another approach, the Dynamic Adaptation Process (DAP) [20], will be used by one project to incorporate implementation considerations in the context of service delivery. Like ADAPT-ITT, DAP engages partners and experts pre- and post-intervention launch and integrates field-based modifications during implementation. Additional participatory approaches will be employed to inform adaptation within PATC^3^H-IN studies, such as crowdsourcing, learning communities, and co-design sessions.

More recently, implementation science experts have been promoting tracking and documenting adaptations to elucidate the mechanisms of action, ensure fidelity to an intervention’s core components, facilitate reproducibility, and strengthen the science of intervention adaptation. To this end, five of the eight teams will rigorously record adaptations using a systematic framework (see Table 6), the Framework for Reporting Adaptations and Modifications to Evidence-based interventions (FRAME) [36, 37] and its companion FRAME-IS. Both approaches document changes to evidence-based practices (EBP) or implementation strategies (respectively) as follows: a description of the EBP or strategy, when and how the modification(s) was made; whether the modification was planned or unplanned; the nature of the content/evaluation/training modification; who made the modification; what was modified; at what level the modification was made; the nature of the content modification; if/how fidelity was impacted by the modification; and rationale/reasons for the modification (including the goal and context) (Table 6).

**Table 6:** Adaptation frameworks

Project	Adaptation Approach	Intervention(s)
ATTUNE	ADAPT-ITT	Peer navigation + LAI ART
iCARE Plus	DAP FRAME-IS	HIV-Treatment: Peer navigation + TXTXT HIV Testing: Social media outreach + peer navigation, PrEP brief intervention
MWOTAJI	FRAME-IS	Malkai Klabu (“Queen Club”), pharmacy-based loyalty program
RISE	ADAPT-ITT, FRAME-IS	HealthMpowerment Digital Health Platform [38]
S-ITEST	Participatory approaches (Crowdsourcing, learning communities) FRAME	4Youth By Youth (4YBY)[39] HIV self-testing kit + photo-verification app or USSD + Linkage to youth-friendly health services
HIP-CY	User-centered design (e.g., focus groups, key informant interviews, community advisory board consultation)	CAB-LA uptake and persistence SEARCH-YOUTH
VS4A	Human-centered design, Crowdsourcing, Co-design workshop, Beta- testing	VITAL Start (Video Intervention to Inspire Treatment Adherence for Life)
ZAIMARA	ADAPT-ITT, FRAME	IMARA-SA

*Notes: ADAPT-ITT*=Assessment, Decision, Adaptation, Production, Topical Experts, Integration, Training, and Testing; *DAP *=Dynamic Adaptation Process; *FRAME-IS *=Framework for Reporting Adaptations and Modifications to Evidence-based Implementation Strategies; *FRAME *=Framework for reporting adaptations and modifications-enhanced; *LAI-ART*=Long-Acting Injectable Antiretroviral Therapy; *CAB-LA*=Cabotegravir Long-Acting ART

### Youth engagement

A key component of PATC^3^H-IN is the involvement of AYA as partners and not solely as research subjects. Each CRC engaged youth from their local setting to inform the development of the research protocol, and most CRCs worked with youth advisory boards as the study was developed. Each CRC will use their own strategies to engage AYA, including participatory approaches such as crowdsourcing, establishing youth advisory boards, and human-centered designs. The levels of engagement will vary across projects, and the extent and how the studies engage AYA will be evaluated and reported in subsequent publications. Importantly, in 2024, the network assembled a PATC^3^H-IN network-level youth advisory board with representatives from each CRC to guide network activities (see Table 1).

## Discussion

The PATC^3^H-IN network was launched in 2023 to spur innovative implementation science research in HIV prevention, care, and treatment for AYA living in LMICs. HIV research has led to life changing prevention and treatment options, but these advances have been slow to reach youth populations in Africa, the region with the highest HIV prevalence. This gap has led to poorly understood complexities that characterize adolescence in LMICs, impeding youth specific HIV programming required for better health outcomes. By establishing CRCs in highly impacted areas, the network offers unique opportunities to grow, innovate, and sustain the next generation of implementation science for young people worldwide, and address an important gap in the current research landscape.

The PATC^3^H-IN network is the first explicit attempt to stimulate much-needed implementation science research to prevent new HIV infections among AYA, and to identify, link, retain in care, and achieve long-term viral suppression across six geographically dispersed countries in sub-Saharan Africa. A recent scoping review on the use of implementation science for the prevention and treatment of HIV among AYA initially identified 44 articles, but the authors noted several limitations. Only four publications used an established implementation science framework [40], youth engagement in the research activities was low (12%), and important implementation outcomes were not measured, namely reach, costs, and sustainability [40]. PATC^3^H-IN provides an opportunity to address these limitations by informing the use of frameworks, active youth participation in the research process, and measuring the full range of implementation outcomes.

A strength of implementation science is the recognition that implementation of evidence-based interventions is highly contextual and not “one-size-fits-all”. Understanding what, when, how, and who should implement interventions depends heavily on the context, including but not limited to the organizational capacity to deliver programs, leadership support for new interventions, and external influences like funding on implementation. Some of these factors are more or less amenable to change and policy action to strengthen uptake, adoption, and sustainability. PATC^3^H-IN was designed with these goals in mind. Investigators were required to engage organizations willing to increase their capacity, partner with country leadership to amplify buy-in, and work with local policy makers to translate effective programs into policy.

The PATC^3^H-IN network will pool implementation science data across sites, constituting one of the largest samples of 12–24-year-olds affected by HIV living in sub-Saharan Africa. While some of the instruments will be tailored to the study’s unique culture, context, population, and partners, they retain the core concepts intended for measurement (e.g., adoption, reach). We will evaluate how well the tools capture adaptations, scale-up, and sustainability of best practices for AYA over time. Furthermore, given scarce resources and the range of competing demands in many African settings, prioritizing the goals will expedite the translation of promising findings into practice for AYA in the region. Additional contributions of the PATC^3^H-IN network are noteworthy.

### Locally-validated implementation frameworks

Most implementation science theories and frameworks were developed by and for research conducted in the United States. Their application to non-Western and resource-constrained settings is questionable. The PATC^3^H-IN studies will use combinations of theories and frameworks to capture the unique circumstances in non-US settings. Projects will draw on determinants, processes, and evaluation models and seek feedback from youth, community members, and other key partners to ensure they are locally-informed. Additionally, investigators will carefully evaluate the utility of the theories and frameworks guiding their projects to strengthen their relevance across African settings. Such data will help future research apply more reliable and valid models and be poised to drive future implementation science research in resource-limited settings.

### Documenting adaptations

The PATC^3^H-IN projects will address a major gap in current implementation research – carefully documenting adaptations to existing evidence-based interventions and implementation strategies to promote scientific rigor during local tailoring. Most PATC^3^H-IN studies will use a systematic approach to record their adaptations, such as ADAPT-ITT [41], FRAME [42], and FRAME-IS [43]. Given the diversity of evidence-based interventions to be tested and the different settings and populations, careful documentation will reveal whether the adaptation process can be generalized. The FRAME will also elucidate important aspects of the process, including who, what, when, and where the modifications were conducted, and these will guide future research.

### Data harmonization

Data harmonization, also referred to as common data elements, is increasingly important to enable comparisons across studies and strengthen confidence in study findings [13]. A benefit of research networks over a single investigation is the cross-adoption of similar tools to measure comparable constructs. Networks can strengthen hypothesis testing by combining smaller samples to increase power and focus on participants who are traditionally hard-to-reach. Research findings are similarly enhanced by testing questions across cultures and contexts. Building on the processes developed in PATC^3^H [13], PATC^3^H-IN investigators reviewed several tools to document the adaptation, delivery, scale-up and sustainability of implementation strategies for AYA affected by HIV in LMICs. Investigators agreed to a set of common measures across five domains – demographics, mental health and substance use, the HIV care continuum, implementation science, and cost effectiveness. These data will promote consistent measurement and cross-study analyses of important research questions best answered by a network of studies (e.g., prevalence of depression across populations). Likewise, relatively little is known about the psychometric properties of implementation science measures within the African region. Cross-study data will inform a test of the reliability and validity of measures across diverse populations, settings, and contexts, addressing the need for contextually relevant implementation research tools [40]. 

### Implementation strategies

The PATC^3^H-IN network is particularly poised to evaluate varied implementation strategies, addressing a significant gap in the science of HIV and AYA. Each study team reported which ERIC implementation strategies they plan to employ [44]. Some strategies were more frequently reported than others, underscoring the greater relevance of specific strategies in LMICs. For example, every project endorsed comprehensive plans for local community engagement to ensure intervention fit. This supports extensive literature that underscores the importance of community engagement in directing how research is conducted, translated, and applied in real settings (e.g., material development, recruitment and retention, intervention delivery, manuscript preparation) [45], especially when applying an intervention developed in a high-income setting to a low-resource environment. Community engagement strengthens reach, feasibility, acceptability, appropriateness, and sustainability [46]. The recent addition of an Implementation Science Coordinating Center to the PATC^3^H-IN will carefully track and monitor implementation strategies and outcomes across network studies. This centralized repository will facilitate secondary analyses of which implementation strategies or combination of strategies optimizes implementation and clinical outcomes.

### Mechanisms of change

Research on the impact of implementation determinants (i.e., barriers, facilitators, constraints) is vast and detailed, but few studies have articulated the mechanisms of change and the processes and core functions that influence implementation [47, 48]. Mechanisms of change exist at multiple levels, and the PATC^3^H-IN studies reflect this diversity, ranging from individual-level mechanisms to broader health-care system influences. Understanding the mechanisms of change will guide the selection of implementation strategies to amplify the reach, uptake, adoption, and sustainability of evidence-based interventions [48]. 

### Global implementation reciprocation or reverse innovation

A common misconception in global health research is that information and expertise flow one-way, from high-resource settings to resource-constrained settings. In fact, mutual sharing of experiences, methodologies, and best practices is equally beneficial to higher-income contexts [49]. For example, resource-constrained environments are often tasked to do more with less. They must innovate in the context of limited resources. Community-health worker outreach is an example of such an innovation driven initially by necessity. In sub-Saharan Africa, many HIV care clinics hired community health workers to dispense ART, because they had few trained clinicians [50]. While initially a response to limited resources, research demonstrated the efficacy of this approach in maximizing adherence and containing costs [51–53]. Now, many programs in the United States use community members to support ART adherence. This type of reverse innovation can address system inequities, build local capacity, and maximize programming for greater efficiency [49]. Building reciprocal partnerships is essential for global health, and represents a paradigm shift towards decolonizing global health, where knowledge production is mutually beneficial [49, 54]. The activities of the CRCs in the PATC^3^H-IN network can inform mutual learning, allowing for reciprocal innovation [49, 54]. 

### Limitations

As with most networks comprised of individually-driven studies, the potential for missing data exists and may prevent secondary analyses that include the full network projects. Still, data collection tools across studies are robust and designed to catch missing data early and address it in real time. Although teams agreed to a set of common data elements, it is possible that not all teams will employ them consistently or at the same time points. Further, individual study designs and data collection processes may limit the depth of information obtained on implementation science strategies, outcomes, or mechanisms of change. Finally, recent executive orders by the Trump administration may impede planned project activities, including the timing of study roll out. This remains to be seen as the courts contend with grant terminations and grant-making restrictions. Nonetheless, PATC^3^H-IN remains a unique opportunity to advance implementation science for AYA affected by HIV living in sub-Saharan Africa.

## Conclusions

We must close the gap between discovery and application of evidence-based interventions in the real world to realize the greatest health benefits of research investments. Implementation science has the potential to achieve this goal through rigorous, locally tailored, and contextually driven research that elucidates how to deliver interventions that maximize reach and scale-up, increase acceptability, ensure fidelity, and optimize sustainability. NIH-funded networks like PATC^3^H-IN offer significant opportunities to inform efficient, effective, and generalizable implementation strategies by testing hypotheses with larger and more diverse populations, conducting secondary analyses of common data elements, and contributing much-needed new information about AYA affected by HIV in sub-Saharan Africa. Findings will yield scientific advances in locally tailored implementation science frameworks for resource-constrained settings; meticulous documentation about the process of adapting interventions for new contexts; rates of mental health distress across contexts; and the costs of intervention delivery in resource-limited settings. A network of studies like PATC^3^H-IN can spearhead new research in reverse innovation by tracking and reporting mutual learning across environments and regions. Together, the studies will identify the factors that expand the reach, adoption, and sustainability of evidence-based HIV interventions across the prevention and care continuum (i.e., HIV prevention, linkage to care, treatment, and support interventions). Similarly, the network provides extensive opportunities for capacity development within projects, across studies, and throughout sub-Saharan Africa and the United States. Shared trainings in implementation science across CRCs can spark new collaborations and additional investment in the next generation of early-career investigators. Finally, PATC^3^H-IN investigators and staff will share best practices while learning from one another in real time about implementation challenges and successes, and problem-solving together to optimize implementation and health outcomes. All of these activities will move us closer to ending the HIV epidemic.

## Supplementary Information

Supplementary Material 1

Supplementary Material 2

## Data Availability

The data and materials used during the current study are available from the corresponding author on reasonable request.
