# Supplementary material for: Strengthening implementation science research to improve adolescent and young adult HIV-prevention and care in Sub-Saharan Africa: PATC3H-IN
Source: BMC Public Health. 2025 Nov 26;25:4333. doi: 10.1186/s12889-025-24995-0 (PMC12750728; doi:10.1186/s12889-025-24995-0)
Supplement: Supplementary file 1 — Supplementary Material 1 [file 12889_2025_24995_MOESM1_ESM.docx]

**Supplementary File**

| **Supplementary Table 1. Implementation Strategies Across the CRCs** | | | | | | | | | |
| --- | --- | --- | --- | --- | --- | --- | --- | --- | --- |
|  | **ATTUNE** | **ICARE PLUS** | **HIP- CY** | **MWOTAJI** | **RISE** | **S-ITEST** | **VS4A** | **ZAIMARA** | TOTAL |
| 1. Use advisory boards and workgroups |  |  |  |  |  |  |  |  | 7 |
| 1. Develop educational materials |  |  |  |  |  |  |  |  | 7 |
| 1. Intervene with patients/consumers to enhance uptake and adherence |  |  |  |  |  |  |  |  | 7 |
| 1. Conduct educational meetings |  |  |  |  |  |  |  |  | 6 |
| 1. Conduct local consensus discussions |  |  |  |  |  |  |  |  | 6 |
| 1. Involve patients/consumers and family members |  |  |  |  |  |  |  |  | 6 |
| 1. Build a coalition |  |  |  |  |  |  |  |  | 6 |
| 1. Assess for readiness and identify barriers and facilitators |  |  |  |  |  |  |  |  | 6 |
| 1. Conduct local needs assessment |  |  |  |  |  |  |  |  | 5 |
| 1. Conduct ongoing training |  |  |  |  |  |  |  |  | 5 |
| 1. Obtain and use patients/consumers and family feedback |  |  |  |  |  |  |  |  | 5 |
| 1. Provide ongoing consultation |  |  |  |  |  |  |  |  | 5 |
| 1. Promote adaptability |  |  |  |  |  |  |  |  | 5 |
| 1. Develop academic partnerships |  |  |  |  |  |  |  |  | 4 |
| 1. Identify and prepare champions |  |  |  |  |  |  |  |  | 4 |
| 1. Prepare patients/consumers to be active participants |  |  |  |  |  |  |  |  | 4 |
| 1. Provide local technical assistance |  |  |  |  |  |  |  |  | 4 |
| 1. Stage implementation scale-up |  |  |  |  |  |  |  |  | 4 |
| 1. Capture and share local knowledge |  |  |  |  |  |  |  |  | 4 |
| 1. Create a learning collaborative |  |  |  |  |  |  |  |  | 3 |
| 1. Tailor strategies |  |  |  |  |  |  |  |  | 3 |
| 1. Use train-the-trainer strategies |  |  |  |  |  |  |  |  | 3 |
| 1. Change service sites |  |  |  |  |  |  |  |  | 2 |
| 1. Conduct educational outreach visits |  |  |  |  |  |  |  |  | 2 |
| 1. Create new clinical teams |  |  |  |  |  |  |  |  | 2 |
| 1. Distribute educational materials |  |  |  |  |  |  |  |  | 2 |
| 1. Facilitation |  |  |  |  |  |  |  |  | 2 |
| 1. Inform local opinion leaders |  |  |  |  |  |  |  |  | 2 |
| 1. Make training dynamic |  |  |  |  |  |  |  |  | 2 |
| 1. Obtain formal commitments |  |  |  |  |  |  |  |  | 2 |
| 1. Provide clinical supervision |  |  |  |  |  |  |  |  | 2 |
| 1. Use data experts |  |  |  |  |  |  |  |  | 2 |
| 1. Audit and provide feedback |  |  |  |  |  |  |  |  | 2 |
| 1. Change physical structure and equipment |  |  |  |  |  |  |  |  | 1 |
| 1. Centralize technical assistance |  |  |  |  |  |  |  |  | 1 |
| 1. Conduct cyclical small tests of change |  |  |  |  |  |  |  |  | 1 |
| 1. Facilitate relay of clinical data to providers |  |  |  |  |  |  |  |  | 1 |
| 1. Visit other sites |  |  |  |  |  |  |  |  | 1 |
| 1. Develop an implementation glossary |  |  |  |  |  |  |  |  | 1 |
| 1. Identify early adopters |  |  |  |  |  |  |  |  | 1 |
| 1. Use an implementation advisor |  |  |  |  |  |  |  |  | 1 |
| 1. Shadow other experts |  |  |  |  |  |  |  |  | 1 |
| 1. Change accreditation or membership requirements |  |  |  |  |  |  |  |  | 1 |
| 1. Change record systems |  |  |  |  |  |  |  |  | 1 |
| 1. Create or change credentialing and/or licensure standards |  |  |  |  |  |  |  |  | 1 |
| 1. Develop a formal implementation blueprint |  |  |  |  |  |  |  |  | 1 |
| 1. Develop and implement tools for quality monitoring |  |  |  |  |  |  |  |  | 1 |
| 1. Develop and organize quality monitoring systems |  |  |  |  |  |  |  |  | 1 |
| 1. Involve executive boards |  |  |  |  |  |  |  |  | 1 |
| 1. Promote network weaving |  |  |  |  |  |  |  |  | 1 |
| 1. Recruit, designate, and train for leadership |  |  |  |  |  |  |  |  | 1 |
| 1. Revise professional roles |  |  |  |  |  |  |  |  | 1 |
| 1. Use data warehousing techniques |  |  |  |  |  |  |  |  | 1 |
| 1. Use other payment schemes |  |  |  |  |  |  |  |  | 1 |
