# Supplementary material for: Strengthening implementation science research to improve adolescent and young adult HIV-prevention and care in Sub-Saharan Africa: PATC3H-IN
Source: BMC Public Health. 2025 Nov 26;25:4333. doi: 10.1186/s12889-025-24995-0 (PMC12750728; doi:10.1186/s12889-025-24995-0)
Supplement: Supplementary file 2 — Supplementary Material 2 [file 12889_2025_24995_MOESM2_ESM.docx]

**Strengthening Implementation Science Research to Improve Adolescent and Young Adult HIV-Prevention and Care in Sub-Saharan Africa: PATC^3^H-IN**

Geri R. Donenberg^1^

Ucheoma Nwaozuru^2^

Sylvia Adebajo^3,4^

Joseph D. Tucker^5,6^

Juliet Iwelunmor^7^

Lisa M. Kuhns^8,9^

Brenda Gati Mirembe^10^

Lisa Hightow-Weidman^11^

Brian Zanoni^12^

Tapiwa A. Tembo^13^

Maria Felix Lupogo^14^

Franklin Yates^15^

**Affiliations:**

1. Center for Dissemination and Implementation Science, Department of Medicine, University of Illinois Chicago, Chicago, Illinois, USA
2. Department of Implementation Science, Wake Forest University School of Medicine, Winston-Salem, North Carolina, USA
3. Institute of Human Virology, University of Maryland, Baltimore, USA
4. Institute of Human Virology, Nigeria
5. Clinical Research Department, Faculty of Infectious and Tropical Diseases, London School of Hygiene and Tropical Medicine, LSHTM, London, England
6. Institute for Global Health and Infectious Diseases, University of North Carolina at Chapel Hill, Chapel Hill, North Carolina, USA
7. Division of Infectious Diseases, Washington University School of Medicine in St. Louis, Saint Louis, Missouri, USA
8. Division of Adolescent Medicine, Ann & Robert H. Lurie Children’s Hospital, Chicago, Illinois, USA
9. Department of Pediatrics, Feinberg School of Medicine, Northwestern University, Chicago, Illinois, USA
10. MU-JHU Research Collaboration, Kampala, Uganda
11. College of Nursing, Florida State University, Florida, USA
12. Department of Pediatrics, Emory University School of Medicine, Georgia, USA
13. Baylor College of Medicine Children's Foundation Malawi, Lilongwe, Malawi
14. Health for a Prosperous Nation, Dar es Salaam, Tanzania
15. Eunice Kennedy Shriver National Institute of Child Health and Human Development, National Institutes of Health, DC, USA

**Corresponding Author:**

Geri R. Donenberg

Center for Dissemination and Implementation Science

Department of Medicine

University of Illinois Chicago

Email: geri.donenberg@nih.gov

**Abstract**

**Background:** Despite significant progress in HIV prevention and treatment, uptake of evidence-based interventions among adolescents and young adults (AYA), particularly in low- and middle-income countries (LMICs), remains low. Implementation research can optimize strategies to enhance reach, uptake, and equitable access to these innovations. The Prevention and Treatment through a Comprehensive Care Continuum for HIV-affected Adolescents in Resource-Constrained Settings Implementation Science Network (PATC^3^H-IN) leverages implementation science to strengthen the delivery and sustainability of evidence-based HIV prevention and care for AYA across six countries in sub-Saharan Africa. This paper outlines PATC^3^H-IN’s goals, summarizes the implementation science (IS) data that will be collected, and highlights the advantages of research networks in advancing science.

**Methods:** The PATC^3^H-IN builds on the existing PATC^3^H consortium to advance IS research targeting AYA in LMICs. The PATC^3^H-IN comprises eight Clinical Research Centers (CRCs) located in Nigeria, Uganda, Malawi, South Africa, Zambia, and Tanzania. Representatives from the CRCs were asked to provide information on the IS components of their proposed studies, including details on study populations, IS frameworks, outcomes, and strategies, mechanisms of change, effectiveness outcomes, and documentation of intervention adaptations. The reports from the CRCs were compared to identify opportunities for advancing science across study sites.

**Results:** The PATC^3^H-IN studies will enroll AYA aged 12-24 years, with some emphasizing key subpopulations, namely AYA living with HIV, sexual and gender minorities, and adolescent girls and young women. All PATC^3^H-IN studies will be guided by one or more implementation science frameworks and theories, with the Reach, Effectiveness, Adoption, Implementation, and Maintenance (RE-AIM) framework most frequently cited (n=4/8). Across the CRCs, 54 unique implementation strategies will be used, with community engagement being the most common. Several studies will document intervention adaptations, and all studies will collect a set of common data elements to facilitate secondary data analyses across projects.

**Conclusions:** The PATC^3^H-IN represents a significant contribution to advancing HIV prevention and care research for AYA in resource-constrained settings. Findings from PATC^3^H-IN will extend our understanding of IS in sub-Saharan Africa, a region particularly burdened by HIV, and for AYA who are traditionally under-represented in IS research.

**Clinical trial number:** Not Applicable

**Keywords:** Adolescents and Young Adults, Implementation Science, HIV Prevention and Care, Evidence-based Interventions, Africa

**Background**

Tremendous achievements have been made since the first AIDS diagnosis in 1981, transforming HIV from an untreatable disease to a fully preventable chronic condition.[[1](#_ENREF_1)] Scientific advancements have led to effective biomedical approaches that could end the epidemic, including formulations that minimize side effects, reduce patient burden, and remain effective for longer periods of time.[[2](#_ENREF_2)] Similarly, new behavioral tools have strengthened prevention [[2](#_ENREF_2), [3](#_ENREF_3)] and improved HIV testing uptake, linkage and retention in care, and adherence to antiretroviral therapy (ART).[[4](#_ENREF_4), [5](#_ENREF_5)] Yet, many of the world’s most vulnerable populations have not benefitted equally from these innovations, in large part a consequence of minimal attention to contextual factors, structural determinants, and system-level barriers at each stage of the HIV-prevention and care continuum, particularly in resource-constrained settings.[[6](#_ENREF_6)]

Implementation science (IS) holds great promise in improving the uptake and adoption of life-saving interventions, but only when evidence-based interventions are tailored for the specific culture, context, and population, engage key partners and communities, employ strategies that address population-specific barriers, and target change mechanisms.[[7](#_ENREF_7)] Unfortunately, efforts to transfer knowledge across situations and settings have been largely disappointing, particularly from high-income to low-to-middle-income environs, between LMICs, and across regions, countries, and implementing partners within sub-Saharan Africa, where the largest number of people have HIV and where adolescents and young adults (AYA) ages 15-24 represent the group at greatest risk of new infections. Despite the high burden of HIV among AYA in this region, HIV testing rates, levels of ART initiation, viral suppression, retention in care, and ART adherence are lower among AYA compared to adults. According to the United Nations, sub-Saharan Africa has the youngest population in the world, with 70% of the population under 30 years old, raising alarms about the future if current HIV incidence rates persist.[[8](#_ENREF_8)] The reasons for the low uptake of evidence-based HIV interventions in lower-income settings are complex, but frequently implicated is the absence of attention to unique contextual barriers, facilitators, and constraints.[[9-11](#_ENREF_9)] Improving uptake and adoption of evidence-based interventions in sub-Saharan Africa is, therefore, among the most important public health challenges if we hope to end HIV as a public health threat.

In direct response to this shifting landscape, *The* *Eunice Kennedy Shriver* National Institute of Child Health and Human Development (NICHD) launched the first *Prevention And Treatment through a Comprehensive Care Continuum for HIV-affected Adolescents in Resource Constrained Settings* (PATC^3^H) consortium comprised of eight research projects implemented across six countries in sub-Saharan Africa and Brazil (see Tucker, Iwelunmor, Abrams, et al., 2021,[[12](#_ENREF_12)] for a full description). Briefly, the original PATC^3^H network focused on implementing and evaluating interventions to achieve long-term viral suppression among AYA with HIV or to prevent HIV acquisition among AYA who engage in behaviors that place them at risk for HIV.[[12](#_ENREF_12)] The studies utilized innovative combination multilevel interventions to improve AYA health outcomes and to explore implementation-related factors that constrained and/or facilitated evidence-based intervention delivery. [[13](#_ENREF_13)] In 2023, NICHD built on the progress and momentum of the PATC^3^H network. The new PATC^3^H-Implementation Science Network (PATC^3^H-IN) expanded the focus on implementation science and HIV-related research to prevent, diagnose, link to care, and achieve viral suppression among AYA living in sub-Saharan Africa. The PATC^3^H-IN [[14](#_ENREF_14)] emphasizes rigorous implementation science approaches to strengthen the reach, access, adoption, affordability, scale-up, and sustainability of evidence-based interventions by localizing and capacitating research in eight Clinical Research Centers (CRC) across six low- to middle-income countries in sub-Saharan Africa. This paper describes the goals of PATC^3^H-IN identified by the eight study teams, and the cross-study implementation science characteristics, to underscore opportunities for cooperation and mutual learning best achieved through a network of projects.

Several advantages of research conducted as a network of projects go beyond the benefits of a single study. Research networks can advance public health when multiple projects test a similar scientific question simultaneously, and investigators can discuss and learn from one another about potential barriers and constraints. Research networks facilitate cross-study pollination of ideas and methodologies, allowing for innovations in real time. Additionally, discoveries can be implemented and expanded more quickly by other projects. Research networks may guide practice decision-making with greater confidence when findings generalize across populations and settings. Finally, research networks can adopt common data elements and harmonize data collection tools, thereby informing novel measurement approaches, driving the science of instrumentation forward, and contributing new findings about the specific topic being measured.

**Methods**

**PATC^3^H-IN**

PATC³H-IN comprises eight CRCs located in high HIV incidence communities across sub-Saharan Africa. The studies supported by each CRC represent a collaboration among investigators and implementers at each stage of the research process, including understanding, guiding, and evaluating the effectiveness and implementation of evidence-based strategies to prevent new HIV infections among AYA, and/or identify, link, retain, and achieve long-term viral suppression among AYA with HIV. The studies will evaluate diverse interventions (see Table 1).

| **Table 1.** PATC^3^H-IN CRCs (2024 – 2029) | | |
| --- | --- | --- |
| **Project Name and Corresponding Acronyms** | **Implementing Location** | **Project Description** |
| Evaluation of Long-Acting Injectable and Teen clubs in adolescents in South Africa (ATTUNE) | South Africa | This study aims to improve HIV care outcomes among adolescents living with HIV in South Africa by evaluating the effectiveness of peer navigation and long-acting injectable antiretroviral therapy (LA-ART). This will include the use of adolescent-friendly services and peer navigation to optimize behavioral interventions before investigating long-acting injectable antiretroviral therapy for adolescents living with HIV in sub-Saharan Africa. |
| Intensive Combination Approach to Rollback the HIV Epidemic in Nigerian Youth Plus Effectiveness/ Implementation Hybrid Study (iCARE Plus) | Nigeria | This study aims to improve HIV prevention and treatment outcomes among Nigerian youth aged 15–24, particularly young men who have sex with men (YMSM) and young transgender women (YTW). The project will scale out and test effectiveness and implementation outcomes on enhanced versions of the iCARE interventions, which include peer navigation and mHealth strategies to increase: a) HIV case-finding, linkage-to-care, and PrEP uptake among YMSM and YTW, and b) viral load suppression in previously unstudied YLH newly-diagnosed and initiating ART. |
| Implementation Science to Understand and Design Stakeholder-Informed Innovative Interventions to Improve Adolescent and Youth HIV Prevention and Care Continuums in Rural and Urban Uganda (MUJHU) | Uganda | This study aims to evaluate evidence-based HIV prevention and care interventions toimprove implementation outcomes among high-risk AYA (15-24 years) in Uganda. The study will implement context-specific community differentiated service delivery models for Cabotegravir, long-acting antiretroviral (CAB-LA) and evaluate a multi-component intervention comprising of life-stage based assessment and support to increase viral suppression in high-risk AYA with HIV. |
| Making women's options for HIV prevention in Tanzania accessible and joining implementation science capacity building (MWOTAJI) | Tanzania | This project aims to evaluate the Malkia Klabu (“Queen Club”) implementation strategy in Tanzania, a loyalty program designed for and by AGYW that creates youth-friendly pharmacies where AGYW can access HIV prevention and sexual and reproductive health (SRH) services, with strong linkages to facility-based care. |
| Resilient HIV Implementation Science with Sexual and Gender Minority Youths using Evidence Clinical Research Center (RISE) | Nigeria, Kenya, Malawi, Zambia | This project aims to adapt, refine, and implement a digital health platform to support HIV prevention and care continuum among sexual and gender minority youth in Nigeria, Kenya, Malawi, and Zambia. |
| Sustaining Innovative Tools to Expand Youth-Friendly HIV Self-Testing (S-ITEST) | Nigeria | This project aims to use participatory approaches (i.e., crowdsourcing designathons and participatory learning communities) to co-create sustainment strategies for the 4Youth By Youth (4YBY) program. The 4YBY program aims to promote the uptake of HIV self-testing and other preventive services among AYA, 14-24 years. |
| VITAL Start (Video-Intervention to Inspire Treatment Adherence for Life) for Adolescents, a video intervention to improve retention and adherence to ART among adolescents living with HIV (VS4A) | Malawi | This project aims to adapt, assess, and support the scale-up of a tailored, video-based counseling package, VITAL (Video Intervention to Inspire Treatment Adherence for Life) Start for Adolescents (VS4A) for ALHIV. |
| Zambian Informed, Motivated Aware and Responsible Adolescents and Adults (ZAIMARA) | Zambia | This project will evaluate the effectiveness and implementation of a CDC “best-evidence” intervention, Informed Motivated Aware and Responsible Adolescents and Adults (IMARA), recently adapted for South Africa (IMARA-SA) on AGYW HIV testing uptake, STI/HIV incidence, and PrEP uptake. |

**PATC^3^H-IN Network Goals**

All PATC^3^H-IN projects were awarded for five years, beginning in October 2023. At the PATC^3^H-IN kick-off meeting in January 2024, each of the eight study teams from Nigeria (S-ITEST, iCARE Plus), Uganda (HIP-CY), Malawi (VS4A), South Africa (ATTUNE), Zambia (ZAIMARA), Tanzania (MWOTAJI), and one team representing Nigeria, Kenya, Malawi and Zambia (RISE) shared their goals for the network. Attendees discussed synergies before launching the studies. The discussions confirmed the value of implementation science (IS was built into the call for proposals, so all staff saw the value of it), building collaborations across the network (e.g., cross-training opportunities), and the development and adoption of common data elements in recognition of the importance of comparing findings across projects. Teams were represented at the meeting by principal investigators, partners, health ministers, AYA representatives, NICHD and Fogarty program staff, Westat (the NIH-contracted company to assist with logistics), and other research project members. The teams reached consensus on five main goals for the network: 1) Develop collaborations across CRCs; 2) build capacity within CRCs to cultivate the next generation of implementation science scholars; 3) strengthen the *science* of implementation science; 4) amplify the scientific impact of the PATC^3^H-IN network; and 5) establish PATC^3^H-IN as a leader in AYA HIV-prevention and care research. The CRCs also agreed on the important role of youth engagement in their projects. Table 2 describes the network’s planned activities to achieve these goals.

| **Table 2.** Goals for the PATC^3^H-IN Network | |
| --- | --- |
| **Goals** | **Activities** |
| 1) Develop collaborations across CRCs | Create complementary research initiatives  Develop new networks  Define ideas for joint manuscripts  Establish learning collaboratives  Build local, regional, and cross-country alliances  Facilitate South-to-South learning |
| 2) Build capacity within CRCs and train the next generation of implementation science scholars | Train local providers in implementation science  Develop cutting-edge resources  Deliver implementation science curricula across CRCs  Train to use mHealth strategies  Diversify the science pipeline  Provide mentorship for junior scholars  Elevate youth voices from development to dissemination  Create a standing youth advisory board to direct network studies  Provide pilot funding for sub-studies |
| 3) Strengthen the *science* of implementation science | Link implementation strategies to health/implementation outcomes  Collect implementation science measures across studies  Create guidelines for co-design workshops  Grow the literature on effective implementation strategies |
| 4) Amplify PATC^3^H-IN’s scientific impact | Learn to write policy briefs  Translate research findings to inform national and global guidelines  Measure success and impact across the consortium  Align study findings with PEPFAR initiatives and goals  Leverage south-to-south alliances for larger impacts  Adapt studies to different regions  Strengthen strategies for scale-up |
| 5) Be a leader in adolescent HIV prevention and care research | Minimize duplication of studies  Create a data harmonization platform  Pool data for subsequent analyses  Increase scientific efficiency  Share best practices in service delivery  Expose investigators and partners to new ideas  Identify new formats and technologies to strengthen health outcomes  Nimbly address emerging research concerns in adolescent HIV  Define how network projects are addressing adolescent-identified concerns |

Note: CRCs=Clinical Research Centers; PATC^3^H-IN=The Prevention and Treatment through a Comprehensive Care Continuum for HIV-affected Adolescents in Resource-Constrained Settings Implementation Science Network

Further, each team reported on the primary features of their research study, namely the target population, implementation theory or framework(s) guiding their project, implementation outcomes of interest, hypothesized mechanisms of change, planned implementation strategies, and any approach to documenting adaptations of interventions and/or implementation strategies. The remainder of this paper describes the implementation characteristics across the studies, concluding with a discussion about the value of a network in advancing science beyond any single individual project.

**Ethics approval**

This study did not involve humans or human data. Instead, we summarized the implementation data proposed to be collected within the PATC^3^H-IN network studies. The data were obtained from responses from the PATC^3^H-IN network investigators or NIH reporter. Therefore, informed consent was not required for this study and IRB approval was not needed. However, every project presented in the paper has been approved by its local ethics group.

**Results**

**Study Populations**

The PATC^3^H-IN network is focused on diverse populations of AYA from six countries in East, West, and Southern Africa. Study populations span the developmental age range from 12 to 24-years-old and represent key populations most vulnerable to HIV transmission and poor health outcomes associated with HIV. All eight studies will enroll adolescents (10-17-year-olds) and young adults (18–24 years-old). Two studies will focus exclusively on primary HIV prevention, and three studies will target youth with HIV. Three studies will include youth with and without HIV. Four studies will intentionally recruit sexual and gender minorities, and two studies will focus exclusively on adolescent girls and young women (AGYW). All studies will emphasize individual-level behavior change, but three studies will also engage the broader context to strengthen AYA outcomes. Specifically, two will engage primary caregivers (VS4A, ZAIMARA), and two will focus on healthcare facility workers and pharmacists (HIP-CY, MWOTAJI). In total, PATC^3^H-IN will enroll 15,386 adolescents and young adults, constituting one of the largest research samples of young people affected by HIV in sub-Saharan Africa.

| **Table 3.** Study population characteristics | | | | | | | | |
| --- | --- | --- | --- | --- | --- | --- | --- | --- |
| **Study Name** | **HIP-CY** | **i-CARE Plus** | **RISE** | **SI-TEST** | **ATTUNE** | **ZAIMARA** | **VS4A** | **MWOTAJI** |
| **Country** | Uganda | Nigeria | Nigeria  Kenya  Malawi  Zambia | Nigeria | South Africa | Zambia | Malawi | Tanzania |
| **Sample size** | 600 | 6600 | 1500 | 1,216 | 720 | 600 dyads  (1200 ppts) | 1800 | 1750 |
| **10-17 years-old (HIV+)** | Yes | Yes | Yes | Yes | Yes | _ | Yes | _ |
| **10-17 years-old (HIV-)** | Yes | Yes | Yes | Yes | _ | Yes | _ | Yes |
| **18-24 years- old (HIV+)** | Yes | Yes | Yes | Yes | Yes | _ | Yes | _ |
| **18-24 years- old (HIV-)** | Yes | Yes | Yes | Yes | _ | Yes | _ | Yes |
| **Key population** | Yes | YMSM  YWH | SGM | Yes | Not targeted; Not excluded | AGYW | Not targeted; Not excluded | AGYW |
| **Other populations** | Health care providers; Pharmacists | _ | _ | _ | _ | Mother figures enrolled with AGYW | Parents/  guardians; Health care workers | Pharmacy staff; Health facility staff; government stakeholders |

Note: YMSM = Young men who have sex with men; SGM = Sexual and gender minorities; AGYW=Adolescent girls and young women; YWH=Youth with HIV

**Implementation Science Theories and Frameworks**

All PATC^3^H-IN studies will be guided by one or more of the seven established implementation science frameworks and theories described by Nilsen’s (2015) [[15](#_ENREF_15)] taxonomy (i.e., evaluation, determinants, and process). The most common framework that will be used is the Reach, Effectiveness, Adoption, Implementation, Maintenance (RE-AIM) evaluation framework, employed by four of the projects.[[16](#_ENREF_16)] RE-AIM refers to four aspects of intervention implementation within health settings, namely whether it is: “reaching” the target population, “effective” at improving the clinical outcome, “implemented” with fidelity, and “maintained” in the health care system.[[17](#_ENREF_17)] The second most common framework, the Consolidated Framework for Implementation Research (CFIR), will be employed by three studies and describes the determinants of implementation.[[18](#_ENREF_18)] These studies will elucidate the barriers, facilitators, and constraints affecting implementation across the five CFIR domains (intervention characteristics, individual characteristics, outer setting, inner setting, and implementation processes).[[19](#_ENREF_19)] The CFIR will also inform study assessments of implementation outcomes (see below).

Two additional frameworks will guide network projects: the Exploration, Preparation, Implementation, Sustainment (EPIS) framework and the Implementation Research Logic Model (IRLM). EPIS focuses on the process of implementation, describing four stages of implementation and the outer and inner context factors[[20](#_ENREF_20)] that inform the implementation process. Research suggests that the time spent in specific EPIS phases predicts successful implementation of evidence-based interventions.[[21](#_ENREF_21)] The IRLM was developed to strengthen the scientific specification, rigor, reproducibility, and transparency[[22](#_ENREF_22)] of implementation science studies by specifying relationships across implementation determinants, mechanisms of change, implementation strategies, and outcomes.[[7](#_ENREF_7)] The IRLM also helps identify the most relevant implementation strategies to employ based on certain determinants and mechanisms.

**Implementation Science Outcomes**

All PATC^3^H-IN studies will evaluate one or more implementation outcomes based on the Proctor et al., (2011) Implementation Outcomes Framework.[[23](#_ENREF_23)] The absence of validated tools to measure implementation outcomes in LMICs, however, has posed a challenge to the network. It is unclear if measures used in high-income countries can be applied in resource-constrained settings, or if they are missing important outcomes unique to these contexts. Building on the original PATC^3^H consortium’s data harmonization process and common data elements,[[13](#_ENREF_13)] PATC^3^H-IN investigators agreed on an expanded set of implementation outcomes and corresponding measurement approaches to use across the network. Both the quantitative and qualitative measures will be used across the PATC^3^H-IN studies. Notably, all eight projects will examine *Effectiveness*, *Implementation, Sustainability*/*Maintenance* and *Adoption,* seven projects will assess *Reach*, six will evaluate *Feasibility*, *Acceptability*, and *Costing/cost effectiveness.* Additionally, three will measure *Appropriateness* and *Fidelity* and one will employ time and motion studies (see Table 3 for the implementation outcomes measured across the CRCs).

| **Table 4.** Implementation Outcomes | | | | | | | | | |
| --- | --- | --- | --- | --- | --- | --- | --- | --- | --- |
| **Study Name** | **HIP-CY** | **i-CARE Plus** | **RISE** | **SI-TEST** | **ATTUNE** | **ZAIMARA** | **VS4A** | **MWOTAJI** |  |
| **Acceptability** | - | Yes | Yes | Yes | Yes | Yes | Yes | - |  |
| **Adoption** | Yes | Yes | Yes | Yes | Yes | Yes | Yes | Yes |  |
| **Appropriateness** | - | Yes | - | - | - | Yes | - | Yes |  |
| **Costing/Cost effectiveness** | Yes | - | Yes | Yes | Yes | Yes | Yes | - |  |
| **Effectiveness** | Yes | Yes | Yes | Yes | Yes | Yes | Yes | Yes |  |
| **Feasibility** | Yes | Yes | - | Yes | Yes | Yes | Yes | - |  |
| **Fidelity** | Yes | - | - | - | - | Yes | Yes | - |  |
| **Implementation** | Yes | Yes | Yes | Yes | Yes | Yes | Yes | Yes |  |
| **Reach** | - | Yes | Yes | Yes | Yes | Yes | Yes | Yes |  |
| **Sustainability/ Maintenance** | Yes | Yes | Yes | Yes | Yes | Yes | Yes | Yes |  |

**Mechanisms of Change**

As the field of implementation science continues to gain traction, there is a growing emphasis on identifying the mechanisms of change.[[24](#_ENREF_24), [25](#_ENREF_25)] Identifying change mechanisms can guide the selection of implementation strategies that address the barriers, facilitators and constraints to achieve change. PATC^3^H-IN studies will evaluate multi-level mechanisms from the individual to the health system. Five studies will focus on individual-level mechanisms, such as enhancing participant motivation, increasing treatment self-efficacy, addressing mental health symptoms, and improving HIV knowledge. Four studies will address interpersonal-level mechanisms by enhancing effective communication with peers, partners, and parents/guardians, increasing healthy sexual relationships, and strengthening peer support for prevention and care. Two studies will enhance participatory and human-centered design mechanisms. Finally, three projects will emphasize health-system-level mechanisms, namely strengthening connections between clinical staff and youth and engaging pharmacy owners and staff.

**Implementation Strategies**

Study teams listed their planned implementation strategies, and these were categorized according to Powell’s (2015) Expert Recommendations for Implementing Change (ERIC) taxonomy.[[26](#_ENREF_26)] The PATC^3^H-IN studies will employ 54 unique implementation strategies, with study teams proposing a range of 8-37 each. Broadly, the implementation strategies are focused on three areas: community engagement, capacity support, and learning to optimize implementation. The most common implementation strategies planned by the seven study teams are community engagement and capacity support for implementation success. They will engage advisory boards and workgroups, develop educational materials, and intervene with patients/consumers to enhance uptake and adherence. The second most common implementation strategies also emphasize community collaborations, capacity building, and optimizing implementation success by attending closely to cultural context, for example, assessing for readiness, identifying barriers and facilitators, building a coalition, conducting educational meetings and local consensus discussions, and involving patients/consumers and family members. Finally, several ERIC strategies were adopted by just one project, such as centralizing technical assistance, conducting cyclical small tests of change, developing a formal implementation blueprint, promoting network weaving, shadowing other experts, and visiting other sites. Details on the planned implementation strategies are provided in Supplementary File 1.

**Effectiveness Outcomes**

All eight studies will evaluate the intervention’s effectiveness on participant outcomes (see Table 4). For studies of youth with HIV, five will assess the impact on viral suppression, three will examine retention in HIV care, and one will test linkage to care, ART initiation, and ART adherence using a combination of biological specimens and health records. Five projects of youth without HIV will evaluate PrEP uptake, and four will assess PrEP persistence. One will examine linkage to PrEP care, and another will test PrEP re-start for those who stop and begin again. Three of these projects will calculate HIV testing uptake and HIV incidence. Three studies will examine sexual behaviors that contribute to the spread of HIV, including self-reported condomless sex, multiple partners, sex with high-risk partners, and positive tests of sexually transmitted infections. All of these are indicators of risk for HIV acquisition. Importantly, all primary prevention studies will assess attitudes and/or perceptions of oral and injectable PrEP to inform future studies of uptake and adoption of long-acting regimens, including both Cabotegravir [[27](#_ENREF_27)] and Lenacapavir.[[28](#_ENREF_28)]

| **Table 5.** Effectiveness Outcomes | | |
| --- | --- | --- |
| **HIV-Continuum** | **Effectiveness Outcomes** | **Studies** |
| **HIV-care and treatment continuum** | Viral suppression | ATTUNE, iCARE Plus, HIP-CY, RISE, VS4A |
|  | Retention in care | ATTUNE, RISE, VS4A |
|  | Linkage to care | iCARE Plus |
|  | ART adherence | iCARE Plus, HIP-CY |
|  | ART initiation | RISE |
| **HIV-prevention continuum** | HIV-risk behavior | ATTUNE, iCARE Plus, S-ITEST, ZAIMARA, HIP-CY |
|  | HTC testing uptake | iCARE Plus, S-ITEST, RISE, ZAIMARA |
|  | Linkage to PrEP | iCARE Plus, S-ITEST |
|  | PrEP uptake | iCARE Plus, HIP-CY, RISE, SI-TEST, ZAIMARA, MWOTAJI |
|  | PrEP persistence | HIP-CY, MWOTAJI, RISE, ZAIMARA |
|  | PrEP restart | RISE, MWOTAJI |
|  | HIV incidence | iCARE Plus, RISE, ZAIMARA, HIP-CY |
|  | STI incidence | ZAIMARA |

**Adaptation of Evidence-Based Interventions and Strategies**

A key component of implementation science emphasizes ensuring fit between an intervention and the target population, setting, and context.[[29](#_ENREF_29)] Accordingly, six of eight PATC^3^H-IN projects will systematically adapt (either pre- or post-launch) an evidence-based intervention to the local contexts and populations where they will be delivered (see Table 5). Most projects will undergo pre-intervention adaptations using participatory and/or user-centered approaches that include local partners (e.g., youth, adults, and community advisory boards). The most common pre-intervention adaptation framework used in three studies is the ADAPT-ITT model, a pragmatic approach to adapting interventions developed in the United States to international settings.[[30-34](#_ENREF_30)] The eight-step framework promotes a combination of formative research to determine local capacity and available resources, plus real-time input from the target population (e.g., theater testing), partners and experts, and pilot testing to create the final intervention.[[35](#_ENREF_35)] Another approach, the Dynamic Adaptation Process (DAP),[[20](#_ENREF_20)] will be used by one project to incorporate implementation considerations in the context of service delivery. Like ADAPT-ITT, DAP engages partners and experts pre- and post-intervention launch and integrates field-based modifications during implementation. Additional participatory approaches will be employed to inform adaptation within PATC^3^H-IN studies, such as crowdsourcing, learning communities, and co-design sessions.

More recently, implementation science experts have been promoting tracking and documenting adaptations to elucidate the mechanisms of action, ensure fidelity to an intervention’s core components, facilitate reproducibility, and strengthen the science of intervention adaptation. To this end, five of the eight teams will rigorously record adaptations using a systematic framework (see Table 5), the Framework for Reporting Adaptations and Modifications to Evidence-based interventions (FRAME)[[36](#_ENREF_36), [37](#_ENREF_37)] and its companion FRAME-IS. Both approaches document changes to evidence-based practices (EBP) or implementation strategies (respectively) as follows: a description of the EBP or strategy, when and how the modification(s) was made; whether the modification was planned or unplanned; the nature of the content/evaluation/training modification; who made the modification; what was modified; at what level the modification was made; the nature of the content modification; if/how fidelity was impacted by the modification; and rationale/reasons for the modification (including the goal and context).

| **Table 6.** Adaptation Frameworks | | |
| --- | --- | --- |
| **Project** | **Adaptation Approach** | **Intervention(s)** |
| ATTUNE | ADAPT-ITT | Peer navigation + LAI ART |
| iCARE Plus | DAP  FRAME-IS | HIV-Treatment: Peer navigation + TXTXT  HIV Testing: Social media outreach + peer navigation, PrEP brief intervention |
| MWOTAJI | FRAME-IS | Malkai Klabu (“Queen Club”), pharmacy-based loyalty program |
| RISE | ADAPT-ITT, FRAME-IS | HealthMpowerment Digital Health Platform [[38](#_ENREF_38)] |
| S-ITEST | Participatory approaches (Crowdsourcing, learning communities)  FRAME | 4Youth By Youth (4YBY)[[39](#_ENREF_39)]  HIV self-testing kit + photo-verification app or USSD + Linkage to youth-friendly health services |
| HIP-CY | User-centered design (e.g., focus groups, key informant interviews, community advisory board consultation) | CAB-LA uptake and persistence  SEARCH-YOUTH |
| VS4A | Human-centered design, Crowdsourcing, Co-design workshop, Beta- testing | VITAL Start (Video Intervention to Inspire Treatment Adherence for Life) |
| ZAIMARA | ADAPT-ITT, FRAME | IMARA-SA |

Notes: ADAPT-ITT= Assessment, Decision, Adaptation, Production, Topical Experts, Integration, Training, and Testing; DAP=Dynamic Adaptation Process; FRAME-IS = Framework for Reporting Adaptations and Modifications to Evidence-based Implementation Strategies; FRAME = Framework for reporting adaptations and modifications-enhanced ; LAI-ART= Long-Acting Injectable Antiretroviral Therapy; CAB-LA= Cabotegravir Long-Acting

**Youth Engagement**

A key component of PATCH^3^H-IN is the involvement of AYA as partners and not solely as research subjects. Each CRC engaged youth from their local setting to inform the development of the research protocol, and most CRCs worked with youth advisory boards as the study was developed. Each CRC will use their own strategies to engage AYA, including participatory approaches such as crowdsourcing, establishing youth advisory boards, and human-centered designs. The levels of engagement will vary across projects, and the extent and how the studies engage AYA will be evaluated and reported in subsequent publications. Importantly, in 2024, the network assembled a PATC^3^H-IN network-level youth advisory board with representatives from each CRC to guide network activities (see Table 1).

**Discussion**

The PATC^3^H-IN network was launched in 2023 to spur innovative implementation science research in HIV prevention, care, and treatment for AYA living in LMICs. HIV research has led to life changing prevention and treatment options, but these advances have been slow to reach youth populations in Africa, the region with the highest HIV prevalence. This gap has led to poorly understood complexities that characterize adolescence in LMICs, impeding youth specific HIV programming required for better health outcomes. By establishing CRCs in highly impacted areas, the network offers unique opportunities to grow, innovate, and sustain the next generation of implementation science for young people worldwide, and address an important gap in the current research landscape.

The PATC^3^H-IN network is the first explicit attempt to stimulate much-needed implementation science research to prevent new HIV infections among AYA, and to identify, link, retain in care, and achieve long-term viral suppression across six geographically dispersed countries in sub-Saharan Africa. A recent scoping review on the use of implementation science for the prevention and treatment of HIV among AYA initially identified 44 articles, but the authors noted several limitations. Only four publications used an established implementation science framework [[40](#_ENREF_40)], youth engagement in the research activities was low (12%), and important implementation outcomes were not measured, namely reach, costs, and sustainability.[[40](#_ENREF_40)] PATC^3^H-IN provides an opportunity to address these limitations by informing the use of frameworks, active youth participation in the research process, and measuring the full range of implementation outcomes.

A strength of implementation science is the recognition that implementation of evidence-based interventions is highly contextual and not “one-size-fits-all”. Understanding what, when, how, and who should implement interventions depends heavily on the context, including but not limited to the organizational capacity to deliver programs, leadership support for new interventions, and external influences like funding on implementation. Some of these factors are more or less amenable to change and policy action to strengthen uptake, adoption, and sustainability. PATC^3^H-IN was designed with these goals in mind. Investigators were required to engage organizations willing to increase their capacity, partner with country leadership to amplify buy-in, and work with local policy makers to translate effective programs into policy.

The PATC^3^H-IN network will pool implementation science data across sites, constituting one of the largest samples of 12–24-year-olds affected by HIV living in sub-Saharan Africa. While some of the instruments will be tailored to the study’s unique culture, context, population, and partners, they retain the core concepts intended for measurement (e.g., adoption, reach). We will evaluate how well the tools capture adaptations, scale-up, and sustainability of best practices for AYA over time. Furthermore, given scarce resources and the range of competing demands in many African settings, prioritizing the goals will expedite the translation of promising findings into practice for AYA in the region. Additional contributions of the PATC^3^H-IN network are noteworthy.

**Locally-validated implementation frameworks**. Most implementation science theories and frameworks were developed by and for research conducted in the United States. Their application to non-Western and resource-constrained settings is questionable. The PATC^3^H-IN studies will use combinations of theories and frameworks to capture the unique circumstances in non-US settings. Projects will draw on determinants, processes, and evaluation models and seek feedback from youth, community members, and other key partners to ensure they are locally-informed. Additionally, investigators will carefully evaluate the utility of the theories and frameworks guiding their projects to strengthen their relevance across African settings. Such data will help future research apply more reliable and valid models and be poised to drive future implementation science research in resource-limited settings.

**Documenting adaptations**. The PATC^3^H-IN projects will address a major gap in current implementation research – carefully documenting adaptations to existing evidence-based interventions and implementation strategies to promote scientific rigor during local tailoring. Most PATC^3^H-IN studies will use a systematic approach to record their adaptations, such as ADAPT-ITT [[41](#_ENREF_41)], FRAME [[42](#_ENREF_42)], and FRAME-IS [[43](#_ENREF_43)]. Given the diversity of evidence-based interventions to be tested and the different settings and populations, careful documentation will reveal whether the adaptation process can be generalized. The FRAME will also elucidate important aspects of the process, including who, what, when, and where the modifications were conducted, and these will guide future research.

**Data harmonization**. Data harmonization, also referred to as common data elements, is increasingly important to enable comparisons across studies and strengthen confidence in study findings.[[44](#_ENREF_44)] A benefit of research networks over a single investigation is the cross-adoption of similar tools to measure comparable constructs. Networks can strengthen hypothesis testing by combining smaller samples to increase power and focus on participants who are traditionally hard-to-reach. Research findings are similarly enhanced by testing questions across cultures and contexts. Building on the processes developed in PATC^3^H [[44](#_ENREF_44)], PATC^3^H-IN investigators reviewed several tools to document the adaptation, delivery, scale-up and sustainability of implementation strategies for AYA affected by HIV in LMICs. Investigators agreed to a set of common measures across five domains – demographics, mental health and substance use, the HIV care continuum, implementation science, and cost effectiveness. These data will promote consistent measurement and cross-study analyses of important research questions best answered by a network of studies (e.g., prevalence of depression across populations). Likewise, relatively little is known about the psychometric properties of implementation science measures within the African region. Cross-study data will inform a test of the reliability and validity of measures across diverse populations, settings, and contexts, addressing the need for contextually relevant implementation research tools.[[40](#_ENREF_40)]

**Implementation strategies**. The PATC^3^H-IN network is particularly poised to evaluate varied implementation strategies, addressing a significant gap in the science of HIV and AYA. Each study team reported which ERIC implementation strategies they plan to employ.[[45](#_ENREF_45)] Some strategies were more frequently reported than others, underscoring the greater relevance of specific strategies in LMICs. For example, every project endorsed comprehensive plans for local community engagement to ensure intervention fit. This supports extensive literature that underscores the importance of community engagement in directing how research is conducted, translated, and applied in real settings (e.g., material development, recruitment and retention, intervention delivery, manuscript preparation),[[46](#_ENREF_46)] especially when applying an intervention developed in a high-income setting to a low-resource environment. Community engagement strengthens reach, feasibility, acceptability, appropriateness, and sustainability.[[47](#_ENREF_47)] The recent addition of an Implementation Science Coordinating Center to the PATC^3^H-IN will carefully track and monitor implementation strategies and outcomes across network studies. This centralized repository will facilitate secondary analyses of which implementation strategies or combination of strategies optimizes implementation and clinical outcomes.

**Mechanisms of change**. Research on the impact of implementation determinants (i.e., barriers, facilitators, constraints) is vast and detailed, but few studies have articulated the mechanisms of change and the processes and core functions that influence implementation.[[48](#_ENREF_48), [49](#_ENREF_49)] Mechanisms of change exist at multiple levels, and the PATC^3^H-IN studies reflect this diversity, ranging from individual-level mechanisms to broader health-care system influences. Understanding the mechanisms of change will guide the selection of implementation strategies to amplify the reach, uptake, adoption, and sustainability of evidence-based interventions.[[49](#_ENREF_49)]

**Global implementation reciprocation or reverse innovation**. A common misconception in global health research is that information and expertise flow one-way, from high-resource settings to resource-constrained settings. In fact, mutual sharing of experiences, methodologies, and best practices is equally beneficial to higher-income contexts.[[50](#_ENREF_50)] For example, resource-constrained environments are often tasked to do more with less. They must innovate in the context of limited resources. Community-health worker outreach is an example of such an innovation driven initially by necessity. In sub-Saharan Africa, many HIV care clinics hired community health workers to dispense ART, because they had few trained clinicians.[[51](#_ENREF_51)] While initially a response to limited resources, research demonstrated the efficacy of this approach in maximizing adherence and containing costs.[[52-54](#_ENREF_52)] Now, many programs in the United States use community members to support ART adherence. This type of reverse innovation can address system inequities, build local capacity, and maximize programming for greater efficiency.[[50](#_ENREF_50)] Building reciprocal partnerships is essential for global health, and represents a paradigm shift towards decolonizing global health, where knowledge production is mutually beneficial.[[50](#_ENREF_50), [55](#_ENREF_55)] The activities of the CRCs in the PATC^3^H-IN network can inform mutual learning, allowing for reciprocal innovation.[[50](#_ENREF_50), [55](#_ENREF_55)]

**Limitations**

As with most networks comprised of individually-driven studies, the potential for missing data exists and may prevent secondary analyses that include the full network projects. Still, data collection tools across studies are robust and designed to catch missing data early and address it in real time. Although teams agreed to a set of common data elements, it is possible that not all teams will employ them consistently or at the same time points. Further, individual study designs and data collection processes may limit the depth of information obtained on implementation science strategies, outcomes, or mechanisms of change. Finally, recent executive orders by the Trump administration may impede planned project activities, including the timing of study roll out. This remains to be seen as the courts contend with grant terminations and grant-making restrictions. Nonetheless, PATC^3^H-IN remains a unique opportunity to advance implementation science for AYA affected by HIV living in sub-Saharan Africa.

**Conclusions**

We must close the gap between discovery and application of evidence-based interventions in the real world to realize the greatest health benefits of research investments. Implementation science has the potential to achieve this goal through rigorous, locally tailored, and contextually driven research that elucidates how to deliver interventions that maximize reach and scale-up, increase acceptability, ensure fidelity, and optimize sustainability. NIH-funded networks like PATC^3^H-IN offer significant opportunities to inform efficient, effective, and generalizable implementation strategies by testing hypotheses with larger and more diverse populations, conducting secondary analyses of common data elements, and contributing much-needed new information about AYA affected by HIV in sub-Saharan Africa. Findings will yield scientific advances in locally tailored implementation science frameworks for resource-constrained settings; meticulous documentation about the process of adapting interventions for new contexts; rates of mental health distress across contexts; and the costs of intervention delivery in resource-limited settings. A network of studies like PATC^3^H-IN can spearhead new research in reverse innovation by tracking and reporting mutual learning across environments and regions. Together, the studies will identify the factors that expand the reach, adoption, and sustainability of evidence-based HIV intervention across the prevention and care continuum (i.e, HIV prevention, linkage to care, treatment, and support interventions). Similarly, the network provides extensive opportunities for capacity development within projects, across studies, and throughout sub-Saharan Africa and the United States. Shared trainings in implementation science across CRCs can spark new collaborations and additional investment in the next generation of early-career investigators. Finally, PATC^3^H-IN investigators and staff will share best practices while learning from one another in real time about implementation challenges and successes, while problem-solving together to optimize implementation and health outcomes. All of these activities will move us closer to ending the HIV epidemic.

**Declarations**

**Ethics approval and consent to participate**

This study did not involve humans or human data. Instead, we summarized the implementation data proposed to be collected within the PATC^3^H-IN network studies. The data were obtained from responses from the PATC^3^H-IN network investigators or NIH reporter. Therefore, informed consent was not required for this study. Therefore, no IRB approval was needed. However, every project presented in the paper has been approved by their local ethics group.

**Consent for publication**

Not applicable

**Availability of data and materials**

The data and materials used during the current study are available from the corresponding author on reasonable request

**Competing interests**

The authors have no relevant financial or non-financial interests to disclose. The content is solely the responsibility of the authors and does not necessarily represent the official views of the NIH.

**Funding**

This work was supported by the National Institute of Child Health and Human Development (NICHD). The content is solely the responsibility of the authors and does not necessarily represent the official views of the National Institutes of Health.

**Authors' contributions**

GD: was involved in the conception, design, and write-up of the implementation science sections and finalization of the manuscript.

UN: significantly contributed to the review, formatting, and finalization of the manuscript

SA: contributed to the editing, reference management, formatting and finalization of the manuscript.

JDT: contributed to the conception and design of the initial draft.

JI: contributed to the discussion section

LMK: contributed to writing some sections of the initial draft

BGM: contributed to writing sections of the manuscript

LHW: contributed to the review and editing of the initial draft

BZ: contributed to the editing of the initial draft

TAT: contributed to writing some sections of the initial draft

MFL: reviewed and approved the final manuscript

FY: reviewed and approved the final manuscript

**Acknowledgements**

Research reported in this was supported by the *Eunice Kennedy Shriver* National Institute of Child Health and Human Development (1UG1HD113250; 1UG1HD113160; 1UG1HD113241; 1UG1HD113163; 1UG1HD113162; 7UG1HD113156) and the National Institute on Minority Health and Health Disparities (1UG1MD019436; 1UG1MD019435). The content is solely the responsibility of the authors and does not necessarily represent the official views of the National Institutes of Health. Support in logistical and operational coordination, regulatory infrastructure, and monitoring of non-registrational trials, data systems infrastructure, emerging research infrastructure, and website administration was provided by Westat Inc.

References

1. Deeks SG, Lewin SR, Havlir DV: **The end of AIDS: HIV infection as a chronic disease**. *Lancet* 2013, **382**(9903):1525-1533.

2. Bekker L-G, Wood R: **Strategic planning for the second era of the HIV epidemic**. *The Lancet Public Health* 2024, **9**(4):e208-e209.

3. Kelley CF, Acevedo-Quinones M, Agwu AL, Avihingsanon A, Benson P, Blumenthal J, Brinson C, Brites C, Cahn P, Cantos VD *et al*: **Twice-Yearly Lenacapavir for HIV Prevention in Men and Gender-Diverse Persons**. *N Engl J Med* 2024.

4. Casale M, Carlqvist A, Cluver L: **Recent interventions to improve retention in HIV care and adherence to antiretroviral treatment among adolescents and youth: a systematic review**. *AIDS patient care and STDs* 2019, **33**(6):237-252.

5. Jopling R, Nyamayaro P, Andersen LS, Kagee A, Haberer JE, Abas MA: **A cascade of interventions to promote adherence to antiretroviral therapy in African countries**. *Current HIV/AIDS Reports* 2020, **17**:529-546.

6. Embleton L, Logie CH, Ngure K, Nelson L, Kimbo L, Ayuku D, Turan JM, Braitstein P: **Intersectional stigma and implementation of HIV prevention and treatment services for adolescents living with and at risk for HIV: opportunities for improvement in the HIV continuum in sub-Saharan Africa**. *AIDS and Behavior* 2023, **27**(Suppl 1):162-184.

7. Smith JD, Li DH, Rafferty MR: **The implementation research logic model: a method for planning, executing, reporting, and synthesizing implementation projects**. *Implementation Science* 2020, **15**:1-12.

8. Mulikita JJ: **Young People’s Potential, the Key to Africa’s Sustainable Development**. In*.*: United Nations; 2021.

9. Docherty M, Shaw K, Goulding L, Parke H, Eassom E, Ali F, Thornicroft G: **Evidence-based guideline implementation in low and middle income countries: lessons for mental health care**. *International journal of mental health systems* 2017, **11**:1-16.

10. Ribeiro WS, Grande AJ, Hoffmann MS, Ziebold C, McDaid D, Fry A, Peixoto C, Miranda C, King D, Tomasi CD: **A systematic review of evidence-based interventions for child and adolescent mental health problems in low-and middle-income countries**. *Comprehensive Psychiatry* 2023, **121**:152358.

11. Whitehorn A, Fu L, Porritt K, Lizarondo L, Stephenson M, Marin T, Aye Gyi A, Dell K, Mignone A, Lockwood C: **Mapping clinical barriers and evidence‐based implementation strategies in low‐to‐middle income countries (LMICs)**. *Worldviews on Evidence‐Based Nursing* 2021, **18**(3):190-200.

12. Tucker JD, Iwelunmor J, Abrams E, Donenberg G, Wilson EC, Blachman-Demner D, Laimon L, Taiwo BO, Kuhns LM, John-Stewart GC *et al*: **Accelerating adolescent HIV research in low-income and middle-income countries: evidence from a research consortium**. *Aids* 2021, **35**(15):2503-2511.

13. Donenberg GR, Merrill KG, Obiezu-Umeh C, Nwaozuru U, Blachman-Demner D, Subramanian S, Fournier A, Iwelunmor J: **Harmonizing implementation and outcome data across HIV prevention and care studies in resource-constrained settings**. *Global Implementation Research and Applications* 2022, **2**(2):166-177.

14. [<https://www.patc3h-in.org/>]

15. Nilsen P: **Making sense of implementation theories, models and frameworks**. *Implementation Science* 2015, **10**(1):53.

16. Glasgow RE, Harden SM, Gaglio B, Rabin B, Smith ML, Porter GC, Ory MG, Estabrooks PA: **RE-AIM Planning and Evaluation Framework: Adapting to New Science and Practice With a 20-Year Review**. *Front Public Health* 2019, **7**:64.

17. Glasgow RE, Vogt TM, Boles SM: **Evaluating the public health impact of health promotion interventions: The RE-AIM framework**. *Am J Public Health* 1999, **89**(9):1322-1327.

18. Damschroder LJ, Reardon CM, Widerquist MAO, Lowery J: **The updated Consolidated Framework for Implementation Research based on user feedback**. *Implement Sci* 2022, **17**(1):75.

19. Damschroder LJ, Aron DC, Keith RE, Kirsh SR, Alexander JA, Lowery JC: **Fostering implementation of health services research findings into practice: a consolidated framework for advancing implementation science**. *Implementation Science* 2009, **4**(1):50.

20. Aarons GA, Hurlburt M, Horwitz SM: **Advancing a Conceptual Model of Evidence-Based Practice Implementation in Public Service Sectors**. *Administration and Policy in Mental Health and Mental Health Services Research* 2011, **38**(1):4-23.

21. Saldana L: **The stages of implementation completion for evidence-based practice: protocol for a mixed methods study**. *Implement Sci* 2014, **9**(1):43.

22. Smith JD, Li DH, Rafferty MR: **The Implementation Research Logic Model: a method for planning, executing, reporting, and synthesizing implementation projects**. *Implementation Science* 2020, **15**(1):84.

23. Proctor E, Silmere H, Raghavan R, Hovmand P, Aarons G, Bunger A, Griffey R, Hensley M: **Outcomes for implementation research: conceptual distinctions, measurement challenges, and research agenda**. *Administration and policy in mental health and mental health services research* 2011, **38**:65-76.

24. Lewis CC, Boyd MR, Walsh-Bailey C, Lyon AR, Beidas R, Mittman B, Aarons GA, Weiner BJ, Chambers DA: **A systematic review of empirical studies examining mechanisms of implementation in health**. *Implementation Science* 2020, **15**:1-25.

25. Lewis CC, Klasnja P, Powell BJ, Lyon AR, Tuzzio L, Jones S, Walsh-Bailey C, Weiner B: **From classification to causality: advancing understanding of mechanisms of change in implementation science**. *Frontiers in public health* 2018, **6**:136.

26. Powell BJ, Waltz TJ, Chinman MJ, Damschroder LJ, Smith JL, Matthieu MM, Proctor EK, Kirchner JE: **A refined compilation of implementation strategies: results from the Expert Recommendations for Implementing Change (ERIC) project**. *Implementation science* 2015, **10**:1-14.

27. Orkin C, Oka S, Philibert P, Brinson C, Bassa A, Gusev D, Degen O, García JG, Morell EB, Tan DH: **Long-acting cabotegravir plus rilpivirine for treatment in adults with HIV-1 infection: 96-week results of the randomised, open-label, phase 3 FLAIR study**. *The Lancet HIV* 2021, **8**(4):e185-e196.

28. Tailor MW, Chahine EB, Koren D, Sherman EM: **Lenacapavir: a novel long-acting capsid inhibitor for HIV**. *Annals of Pharmacotherapy* 2024, **58**(2):185-195.

29. Movsisyan A, Arnold L, Evans R, Hallingberg B, Moore G, O'Cathain A, Pfadenhauer LM, Segrott J, Rehfuess E: **Adapting evidence-informed complex population health interventions for new contexts: a systematic review of guidance**. *Implement Sci* 2019, **14**(1):105.

30. Conroy AA, Tebbetts S, Darbes LA, Hahn JA, Neilands TB, McKenna SA, Mulauzi N, Mkandawire J, Ssewamala FM: **Development of an economic and relationship-strengthening intervention for alcohol drinkers living with HIV in Malawi**. *AIDS and Behavior* 2023, **27**(7):2255-2270.

31. Crooks N, Debra A, Coleman D, Sosina W, Singer R, Jeremiah R, Green B, Johnson W, Caldwell C, Patil C: **Application of ADAPT-ITT: adapting an evidence-based HIV/STI mother-daughter prevention intervention for Black male caregivers and girls**. *BMC Public Health* 2023, **23**(1):1426.

32. Sundararajan R, Ponticiello M, Birch G, Nuwagaba G, Alaiku R, Nansera D, Mwanga-Amumpaire J, Muyindike W: **Adaption and pilot testing of a lay HIV supporter program for traditional healers: a mixed methods study in rural Uganda**. *Implementation Science Communications* 2023, **4**(1):87.

33. Tran HV, Nong HT, Tran TT, Filipowicz TR, Landrum KR, Pence BW, Le GM, Nguyen MX, Chibanda D, Verhey R: **Adaptation of a problem-solving program (Friendship Bench) to treat common mental disorders among people living with HIV and AIDS and on methadone maintenance treatment in Vietnam: formative study**. *JMIR formative research* 2022, **6**(7):e37211.

34. Woolf-King SE, Firkey M, Foley JD, Bricker J, Hahn JA, Asiago-Reddy E, Wikier J, Moskal D, Sheinfil AZ, Ramos J: **Development of a telephone-delivered Acceptance and Commitment Therapy intervention for people living with HIV who are hazardous drinkers**. *AIDS and Behavior* 2022, **26**(9):3029-3044.

35. Libous JL, Montanez NA, Dow DE, Kapetanovic S, Buckley J, Kakhu TJ, Kamthunzi P, Maliwichi LA, Vhembo T, Chawana TD *et al*: **IMPAACT 2016: Operationalizing HIV Intervention Adaptations to Inform the Science and Outcomes of Implementation**. *Front Reprod Health* 2021, **3**:662912.

36. Stirman SW, Miller CJ, Toder K, Calloway A: **Development of a framework and coding system for modifications and adaptations of evidence-based interventions**. *Implementation Science* 2013, **8**:1-12.

37. Wiltsey Stirman S, Baumann AA, Miller CJ: **The FRAME: an expanded framework for reporting adaptations and modifications to evidence-based interventions**. *Implementation Science* 2019, **14**:1-10.

38. Hightow-Weidman LB, LeGrand S, Muessig KE, Simmons RA, Soni K, Choi SK, Kirschke-Schwartz H, Egger JR: **A randomized trial of an online risk reduction intervention for young black MSM**. *AIDS and Behavior* 2019, **23**:1166-1177.

39. Iwelunmor J, Tucker JD, Obiezu-Umeh C, Gbaja-Biamila T, Oladele D, Nwaozuru U, Musa AZ, Airhihenbuwa CO, Muessig K, Rosenberg N: **The 4 Youth by Youth (4YBY) pragmatic trial to enhance HIV self-testing uptake and sustainability: study protocol in Nigeria**. *Contemporary clinical trials* 2022, **114**:106628.

40. Vorkoper S, Tahlil KM, Sam-Agudu NA, Tucker JD, Livinski AA, Fernando F, Sturke R: **Implementation science for the prevention and treatment of HIV among adolescents and young adults in sub-Saharan Africa: a scoping review**. *AIDS and Behavior* 2023, **27**(Suppl 1):7-23.

41. Wingood GM, DiClemente RJ: **The ADAPT-ITT model: a novel method of adapting evidence-based HIV Interventions**. *J Acquir Immune Defic Syndr* 2008, **47 Suppl 1**:S40-46.

42. Wiltsey Stirman S, Baumann AA, Miller CJ: **The FRAME: an expanded framework for reporting adaptations and modifications to evidence-based interventions**. *Implement Sci* 2019, **14**(1):58.

43. Miller CJ, Barnett ML, Baumann AA, Gutner CA, Wiltsey-Stirman S: **The FRAME-IS: a framework for documenting modifications to implementation strategies in healthcare**. *Implement Sci* 2021, **16**(1):36.

44. Donenberg GR, Merrill KG, Obiezu-Umeh C, Nwaozuru U, Blachman-Demner D, Subramanian S, Fournier A, Iwelunmor J: **Harmonizing Implementation and Outcome Data Across HIV Prevention and Care Studies in Resource-Constrained Settings**. *Glob Implement Res Appl* 2022, **2**(2):166-177.

45. Powell BJ, Waltz TJ, Chinman MJ, Damschroder LJ, Smith JL, Matthieu MM, Proctor EK, Kirchner JE: **A refined compilation of implementation strategies: results from the Expert Recommendations for Implementing Change (ERIC) project**. *Implementation Science* 2015, **10**(1):21.

46. Han HR, Xu A, Mendez KJW, Okoye S, Cudjoe J, Bahouth M, Reese M, Bone L, Dennison-Himmelfarb C: **Exploring community engaged research experiences and preferences: a multi-level qualitative investigation**. *Res Involv Engagem* 2021, **7**(1):19.

47. Tumwesige W, Namatovu P, Bahar OS, Byansi W, McKay MM, Ssewamala FM: **Engaging community and governmental partners in improving health and mental health outcomes for children and adolescents impacted by HIV/AIDS in Uganda**. *Pediatr Med* 2021, **4**.

48. Leeman J, Birken SA, Powell BJ, Rohweder C, Shea CM: **Beyond “implementation strategies”: classifying the full range of strategies used in implementation science and practice**. *Implementation Science* 2017, **12**:1-9.

49. Aarons GA, Reeder K, Sam-Agudu NA, Vorkoper S, Sturke R: **Implementation determinants and mechanisms for the prevention and treatment of adolescent HIV in sub-Saharan Africa: concept mapping of the NIH Fogarty International Center Adolescent HIV Implementation Science Alliance (AHISA) initiative**. *Implementation science communications* 2021, **2**(1):53.

50. Sors TG, O'Brien RC, Scanlon ML, Bermel LY, Chikowe I, Gardner A, Kiplagat J, Lieberman M, Moe SM, Morales-Soto N *et al*: **Reciprocal innovation: A new approach to equitable and mutually beneficial global health partnerships**. *Glob Public Health* 2023, **18**(1):2102202.

51. Mwai GW, Mburu G, Torpey K, Frost P, Ford N, Seeley J: **Role and outcomes of community health workers in HIV care in sub-Saharan Africa: a systematic review**. *J Int AIDS Soc* 2013, **16**(1):18586.

52. Mukherjee JS, Barry D, Weatherford RD, Desai IK, Farmer PE: **Community-Based ART Programs: Sustaining Adherence and Follow-up**. *Curr HIV/AIDS Rep* 2016, **13**(6):359-366.

53. Kabore I, Bloem J, Etheredge G, Obiero W, Wanless S, Doykos P, Ntsekhe P, Mtshali N, Afrikaner E, Sayed R *et al*: **The effect of community-based support services on clinical efficacy and health-related quality of life in HIV/AIDS patients in resource-limited settings in sub-Saharan Africa**. *AIDS Patient Care STDS* 2010, **24**(9):581-594.

54. Nachega JB, Adetokunboh O, Uthman OA, Knowlton AW, Altice FL, Schechter M, Galarraga O, Geng E, Peltzer K, Chang LW *et al*: **Community-Based Interventions to Improve and Sustain Antiretroviral Therapy Adherence, Retention in HIV Care and Clinical Outcomes in Low- and Middle-Income Countries for Achieving the UNAIDS 90-90-90 Targets**. *Curr HIV/AIDS Rep* 2016, **13**(5):241-255.

55. Vroonen L, Moraes K, Masquillier C, Bastiaens H, Wouters E, De Wet K: **Beyond reverse innovation in healthcare: A step towards global health justice through reciprocity**. *International Health Trends and Perspectives* 2023, **3**(3):383-386.
